# SPAG5 upregulation contributes to enhanced c-MYC transcriptional activity via interaction with c-MYC binding protein in triple-negative breast cancer

**DOI:** 10.1186/s13045-019-0700-2

**Published:** 2019-02-08

**Authors:** Ming Li, Anqi Li, Shuling Zhou, Hong Lv, Wentao Yang

**Affiliations:** 1 Department of Pathology, Fudan University Shanghai Cancer Center, 270 Dongan Road, Shanghai, 200032 China; 2 Department of Oncology, Shanghai Medical College, Fudan University, Shanghai, People’s Republic of China; 3 Institute of Pathology, Fudan University, Shanghai, China

**Keywords:** SPAG5, MYCBP, Olaparib, C-MYC, Triple-negative breast cancer

## Abstract

**Background:**

Triple-negative breast cancer (TNBC) is an aggressive breast cancer subtype that lacks effective therapeutic targets. Sperm-associated antigen 5 (SPAG5) is a mitotic spindle-associated protein that is involved in various biological processes in cervical cancer and bladder urothelial carcinoma. However, the role of SPAG5 in TNBC remains undefined.

**Methods:**

The expression of SPAG5 was examined in TNBC patients via quantitative real-time polymerase chain reaction (qRT-PCR), western blotting, and immunohistochemistry (IHC). The biological functions of SPAG5 in TNBC and the underlying mechanisms were investigated in vitro and in vivo.

**Results:**

SPAG5 expression was significantly upregulated in TNBC tissues compared with that in paired adjacent noncancerous tissues (ANTs). High SPAG5 expression was associated with increased lymph node metastasis and high risk of local recurrence. SPAG5 protein expression was significantly associated with poor disease-free survival in TNBC. Gene set enrichment analysis of TNBC data from The Cancer Genome Atlas (TCGA) indicated that high SPAG5 expression was significantly associated with cell cycle and the ATR-BRCA pathway. Functional assays demonstrated that SPAG5 expression promoted tumor growth in vitro and in vivo. In addition, SPAG5-silenced cells were more sensitive to the PARP inhibitor (PARPi) olaparib. Mechanistically, SPAG5 interacted with c-MYC binding protein (MYCBP), thereby increasing MYCBP protein levels and leading to increased c-MYC transcriptional activity, which promoted the expression of the c-MYC target genes: CDC20, CDC25C, BRCA1, BRCA2, and RAD51.Knockdown of MYCBP or c-MYC abolished the SPAG5-induced cell-cycle progression and cell proliferation of TNBC.

**Conclusions:**

Collectively, our results indict that SPAG5 is an efficient prognostic factor in TNBC, and that SPAG5 knockdown increases the sensitivity of TNBC to the PARPi olaparib. SPAG5 promotes tumor growth and DNA repair by increasing c-MYC transcriptional activity via interaction with MYCBP. The SPAG5/MYCBP/c-MYC axis may represent a potential therapeutic target for TNBC treatment.

**Electronic supplementary material:**

The online version of this article (10.1186/s13045-019-0700-2) contains supplementary material, which is available to authorized users.

## Introduction

Triple-negative breast cancer (TNBC) is the most lethal subtype of breast cancer, and accounts for 15–20% of all breast cancers due to the lack of clinically available targeted therapies [1]. Thus, further elucidating the potential biological mechanisms of TNBC is critical to optimizing clinical outcomes. Notably, compared with other breast cancer subtypes, TNBC shows a higher prevalence of germline breast cancer-associated gene (BRCA) mutations [2, 3] and tumor-infiltrating lymphocytes [4, 5]. Moreover, a recent report demonstrated that DNA repair deficiency and host antitumor immunity offered new potential biomarker-driven approaches to TNBC treatment [6].

Sperm-associated antigen 5 (SPAG5) is a mitotic spindle-associated protein needed for the maintenance of sister chromatid cohesion and centrosome integrity during mitosis [7]. SPAG5 is upregulated in cervical cancer and bladder cancer and implicated in tumorigenesis [8–10]. SPAG5 may also be used as a proliferation marker in early breast cancer [7, 11]. Since SPAG5 plays an essential role in cell cycle progression, SPAG5 inhibition may contribute to chromosome instability and influence sensitivity to chemotherapy. A previous study indicated that high SPAG5 expression in breast cancer patients was an independent predictor of an increased proportion of pathological complete response after receiving a combined cytotoxic chemotherapy, because SPAG5 (Ch17q11.2) is located at CEP17, which is a marker of chromosomal instability and linked to anthracycline sensitivity [7]. In addition, another study in bladder urothelial carcinoma indicated that SPAG5 overexpression conferred resistance to cisplatin [8]. Overall, SPAG5 is suggested to play a pivotal role in tumorigenesis and may be a useful marker of chemotherapy sensitivity. However, to date, the role of SPAG5 in TNBC has not been clarified and whether SPAG5 expression is associated with PARP inhibitor (PARPi) sensitivity remains unknown.

To explore the potential functions of SPAG5 in TNBC, we performed a gene set enrichment analysis (GSEA) using mRNA expression data from The Cancer Genome Atlas (TCGA) database, and the results showed that high SPAG5 expression was significantly correlated with cell-cycle-related genes, G2-phase-related genes, and ATR BRCA pathway-related genes. Previous studies indicated that deficiencies in DNA repair pathways, such as BRCA1 and BRCA2 mutations, can increase PARPi activity in breast and ovarian cancer [12]. The PARPi olaparib (Lynparza, AstraZeneca) has recently been approved for the treatment of ovarian cancers with BRCA1/2 mutations [13]. Thus, SPAG5 expression may be a potential biomarker that confers vulnerability to PARPi in TNBC.

Recently, a study revealed that SPAG5 could interact with c-MYC binding protein (MYCBP) in human cells [14]. MYCBP binds to the N-terminus of the oncogenic protein c-MYC, thereby enhancing the ability of c-MYC to activate E-box-dependent transcription [15]. c-MYC is a crucial oncogenic transcription factor that can regulate many genes involved in multiple cellular processes, including cell growth, cell-cycle control, and DNA repair, and it is highly expressed in basal-like breast cancer [16]. Moreover, a study in TNBC showed that c-MYC could directly upregulate the homologous recombination (HR) DNA repair protein RAD51, which results in resistance to PARPi, whereas inhibition of c-MYC or RAD51 increased PARPi-sensitivity independent of the BRCA status [17]. Therefore, we examined the predictive role of SPAG5 in PARPi therapy and assessed whether the role of SPAG5 in TNBC is mediated through interaction with the MYCBP/c-MYC pathway.

In addition, a previous study analyzed tumor-infiltrating immune cells in RNA-sequencing samples from TCGA and found that SPAG5 is a cancer/testis gene that is positively associated with CD8 T-cell infiltration [18]. Thus, in this study, we also investigated the correlation of SPAG5 with CD8 T-cell infiltration in tumor samples of breast cancer patients.

In the present study, we investigated SPAG5 expression and function and its interaction with MYCBP in TNBC. Taken together, our finds suggested that TNBC patients with high SPAG5 expression have poor prognoses. SPAG5 promoted tumor growth and decreased cell sensitivity to PARPi via the MYCBP/c-MYC pathway in TNBC.

## Methods

### Patients and samples

The study was approved by the Institutional Review Board of the Fudan University Shanghai Cancer Center, and informed consent was obtained from all subjects. SPAG5 expression in breast cancer patients was detected using western blotting, quantitative real-time polymerase chain reaction (qRT-PCR), and immunohistochemistry (IHC). qRT-PCR was performed to examine SPAG5 mRNA expression in 65 paired TNBC tumor samples and adjacent normal tissues (ANTs) collected between 2013 and 2014.Western blotting was performed to examine SPAG5 protein expression in four paired TNBC tumor samples and ANTs. IHC staining for SPAG5, MYCBP, and CD8 expression was performed on a tissue microarray (TMA) with 183 paraffin-embedded samples from patients diagnosed with invasive ductal breast cancer (IDC) collected between 2006 and 2008. SPAG5 expression was correlated with patients’ clinical parameters and prognoses.

### Cell culture and reagents

All cell lines (MDA-MB-231, MDA-MB-468, Hs- 578t, BT20, and BT549) were kindly provided by Dr. Daqiang Li and were maintained according to standard American Type Culture Collection (ATCC) protocols. Mycoplasma contamination was tested using a Universal Mycoplasma Detection Kit (ATCC).

Olaparib (purchased from MCE.) was suspended in dimethyl sulfoxide (DMSO). Cells were seeded in conventional 96-well plates with 100 μl of medium at a concentration of 1 × 10^4^ cells/well. The cells were then cultured for 48 h and tested using a CCK-8 kit.

The antibodies used for FACS were anti-CD24-PE and anti-ESA-APC (Miltenyi Biotec). The antibody for SPAG5 (sc-100,885) western blot analysis was purchased from Santa Cruz. The antibody for SPAG5 (HPA022008) immunohistochemistry (IHC) analysis was purchased from Sigma. Antibodies targeting GAPDH (ab181602), vinculin (ab18058), MYCBP (ab6631), and Ki-67 (ab92742) were purchased from Abcam. Antibodies targeting CD8 (Cell Signaling Technology, CST#70306), Flag (CST#8146), IgG (CST#5415), c-MYC (CST#13987), p27 (CST#3698), p21 (CST#8831), Cyclin B1 (CST#4135), Cyclin A2 (CST#4656), and BRCA1 (CST#9010) were purchased from CST. The BRCA2 (19791-1-AP) antibody was purchased from Proteintech. Antibodies targeting CDC20 (abs135883) and RAD51 (abs137543) were purchased from Absin.

### Immunohistochemistry (IHC)

IHC was performed to assess SPAG5, MYCBP, CD8, and Ki-67 expression as previously described [19]. SPAG5 and MYCBP staining was scored by two pathologists using H scores (ranging between 0 and 300), which are based on the staining intensity and proportion of positively stained cells. In this study, we first estimated and then used the cut-off value calculated with the receiver operating characteristic (ROC) curve to divide patients into groups of high vs. low expression of SPAG5. CD8 staining was scored according to the infiltration density, from 0 (absent) to 3 (dense).

### RNA extraction and quantitative real-time PCR

Cells were collected, and total RNA was extracted using TRIzol reagent (Invitrogen, Carlsbad, CA, USA) according to the manufacturer’s protocol. Reverse transcription (RT) was conducted using PrimeScript RT Master Mix (Takara, Dalian, China) to synthesize cDNA. qRT–PCR was performed with SYBR Green PCR Master Mix (Takara, Dalian, China) on an ABI 7900HT (PE Applied Biosystems) qPCR machine. For relative quantification, target gene mRNA expression was normalized to β-actin expression. The primers used in the qRT-PCR assays are listed in (Additional file 1: Table S1).

### Western blotting

Cells were collected and lysed with RIPA buffer (Thermo Scientific). Proteins were quantified using a bicinchoninic acid assay (Thermo Scientific), resolved by SDS-PAGE, and transferred onto PVDF membranes (Millipore, Billerica, USA). Antibody detection was conducted using an enhanced chemiluminescent substrate kit (Yeasen).

### RNA interference, plasmid transfection, and lentivirus transduction

SPAG5 siRNA and MYCBP shRNA sequences and negative controls were designed by GenePharma (Shanghai, China) and used to transfect cells using Lipofectamine 3000 (Invitrogen) according to the manufacturer’s instructions. To construct stable SPAG5 knockdown cells, shSPAG5 virus and shCtrl were generated. Hs-578t and BT549 cells were infected with the lentivirus and selected with puromycin to establish stable SPAG5 knockdown cells. The target sequences of SPAG5 siRNAs, MYCBP shRNAs, and c-MYC siRNAs are listed in (Additional file 1: Table S1).

For overexpression, full-length SPAG5 was identified and cloned to generate a Flag-SPAG5 construct. To construct stable SPAG5 overexpression cells, lentivirus was constructed and used to infect MDA-MB-231 and MDA-MB-468 cells, which were then selected with puromycin.

### Cell viability and colony-formation assay

Cells were seeded in 96-well plates and cell viability was assessed using a Cell Counting Kit-8 (CCK8; Dojindo, Kumamoto, Japan) according to the manufacturer’s instructions. For colony-formation assays, 1000 viable cells were seeded in 6-well plates and cultured for 2 weeks. Colonies were fixed with ethanol and stained with 1% crystal violet before being counted.

### Flow cytometry analysis

Cell cycle and cell apoptosis analyses were performed via flow cytometry on a FACScan instrument (Beckman Coulter, Brea, CA, USA). For the cell cycle analysis, cells were harvested, fixed with 70% ethanol at 4 °C overnight, and stained with propidium iodide (50 μg/ml), containing 100 μg/ml RNase A for 15 min. For apoptosis analysis, cells were harvested, washed with PBS, and incubated with Annexin V-PE and 7-AAD (BD Biosciences, San Diego, CA, USA).

For quantification of the CD24-ESA+ population, cells were collected, washed with PBS, and incubated with anti-CD24-P, and anti-ESA-APC antibodies for 30 min at 4 °C. Following incubation with antibody, the cells were washed with PBS twice before analysis.

### Mammosphere culture

Single cells were plated at a density of 500 cells /ml in ultralow attachment 24-well plates (Corning). The cells were cultured in DMEM/F12 (Gibco) and supplemented with B27 (Life Technologies), 20 ng/ml epidermal growth factor, 20 ng/ml basic fibroblast growth factor, and 4 mg/ml heparin (Sigma-Aldrich) for 9–12 days. For evaluation, the mammospheres were photographed under an inverted microscope (× 10 objective, Olympus). The mammosphere diameters were analyzed using Image-Pro Plus 6.0 software (Media Cybernetics).

### Tumor xenograft models

All animal studies were approved by the Institutional Animal Care and Use Committee of Shanghai Cancer Center, Fudan University. For tumor formation, 2 × 10^6^ SPAG5 overexpression and vector MDA-MB-231 cells were suspended in 0.2 ml PBS. Six-week-old female BALB/c nude mice were randomly assigned to two groups (*n* = 5 per group), and cells were subsequently injected into the fourth mammary fat pad. Tumor volumes were measured twice a week after the appearance of tumors, and the tumor volume was calculated as (length × width^2^)/2. After 6 weeks, the mice were sacrificed, and the tumors were harvested and analyzed using IHC analyses.

### Transcriptome microarray

The transcriptome profiles of SPAG5 overexpression and vector MDA-MB-231 cells were determined using an Illumina sequencing platform, and RNA integrity was evaluated using an Agilent 2100 Bioanalyzer (Agilent Technologies, Santa Clara, CA, USA). Samples with an RNA integrity number (RIN) ≥ 7 were subjected to subsequent analysis. Libraries were constructed using a TruSeq Stranded mRNA LT Sample Prep Kit (Illumina, San Diego, CA, USA) according to the manufacturer’ s instructions. Raw data (raw reads) were processed using an NGS QC Toolkit. Reads containing poly-N and low quality reads were removed to obtain the clean reads. Then, the clean reads were mapped to the reference genome using hisat2. Fold changes > 1.5 were set as the threshold for significantly differential expression. Hierarchical clustering analysis of differentially expressed genes (DEGs) was performed to explore the gene expression patterns. GO and KEGG pathway enrichment analyses of the DEGs were performed using R based on the hypergeometric distribution.

### Immunofluorescence (IF)

For IF staining, cells were grown on glass coverslips and then fixed with 4% paraformaldehyde for 15 min at room temperature. Following fixation, the cells were permeabilized using 0.5% Triton X-100 for 15 min and blocked with 5% goat serum for 1 h. Then, the cells were incubated with primary antibodies overnight at 4 °C and appropriate secondary antibodies conjugated with Alexa 555 (red) or Alexa 488 (green) (Cell Signaling Technology). DAPI-containing mounting medium (Abcam, Shanghai, China) was used to stain nuclei and mount cells. A Leica SP5 Laser Scanning Confocal Microscope (Leica Microsystems, Buffalo Grove, IL, USA) was used to capture and analyze images.

### Immunoprecipitation (IP)

MDA-MB-231 and BT549 cells infected with Flag-SPAG5 were lysed on ice in NP-40 lysis buffer (50 mM Tris-HCl, pH 8, 150 mM NaCl, 0.5% NP- 40, 10% glycerol, 2 mM MgCl2, and 1 mM EDTA) supplemented with containing 1× protease inhibitor cocktail (Selleck, B14001) and 1× phosphatase inhibitor cocktail (Selleck, B15001). Cell lysates were incubated with 3 μg Flag antibody (Cell Signaling Technology) overnight at 4 °C, followed by precipitation with protein G Sepharose beads (CST#70024). Immuno-complexes were washed before being resolved by SDS-PAGE and detected by western blot using the indicated antibodies.

### Chromatin immunoprecipitation (CHIP)

CHIP assays were performed with nuclear extracts from BT549 siCtrl and siSPAG5 cells using Chromatin Immunoprecipitation Kits (EZ-Magna CHIP™ A/G, Catalog#17–10,086) following the manufacturer’s protocol. Briefly, 1 × 10^7^ cells were fixed with 1% formaldehyde for 10 min at room temperature to cross-link proteins to DNA. After termination of the reaction with glycine for 5 min, the cells were lysed in cell lysis buffer and nuclear lysis buffer, and sonication was performed to shear the chromatin to a manageable size. Then, the cross-linked chromatin was immunoprecipitated with antibody against c-MYC (CST), RNA polymerase II, or IgG. Protein–DNA cross-links were reversed, and the DNA was purified. qRT-PCR was performed with the purified CHIP DNA using primer sequences targeting the specific promoter region of the following genes: CDC20, CDC25C [20], RAD51, BRCA1, and BRCA2. The primers used in the CHIP-qPCR assays are listed in (Additional file 1: Table S1).

### Statistics

Statistical analyses were performed using IBM SPSS 20.1 and GraphPad Prism v 7.0 (GraphPad Software, San Diego, CA, USA). The results are presented as the mean ± standard deviation from at least two independent experiments. Two-tailed Student *t* tests or a one-way ANOVA was applied to assess difference between or among different groups. The chi-square test was used to analyze the relationship between SPAG5 protein expression and clinicopathologic parameters. The Kaplan–Meier method and log-rank test were used for survival analysis. Spearman rank correlation test was used to calculate correlation coefficients, and *p <* 0.05 was considered statistically significant.

## Results

### Increased SPAG5 expression promotes breast cancer progression and is correlated with poor prognosis

The expression of SPAG5 was examined in TNBC tissues using qRT-PCR, western blotting, IHC, and specific databases.

**Fig. 1:**
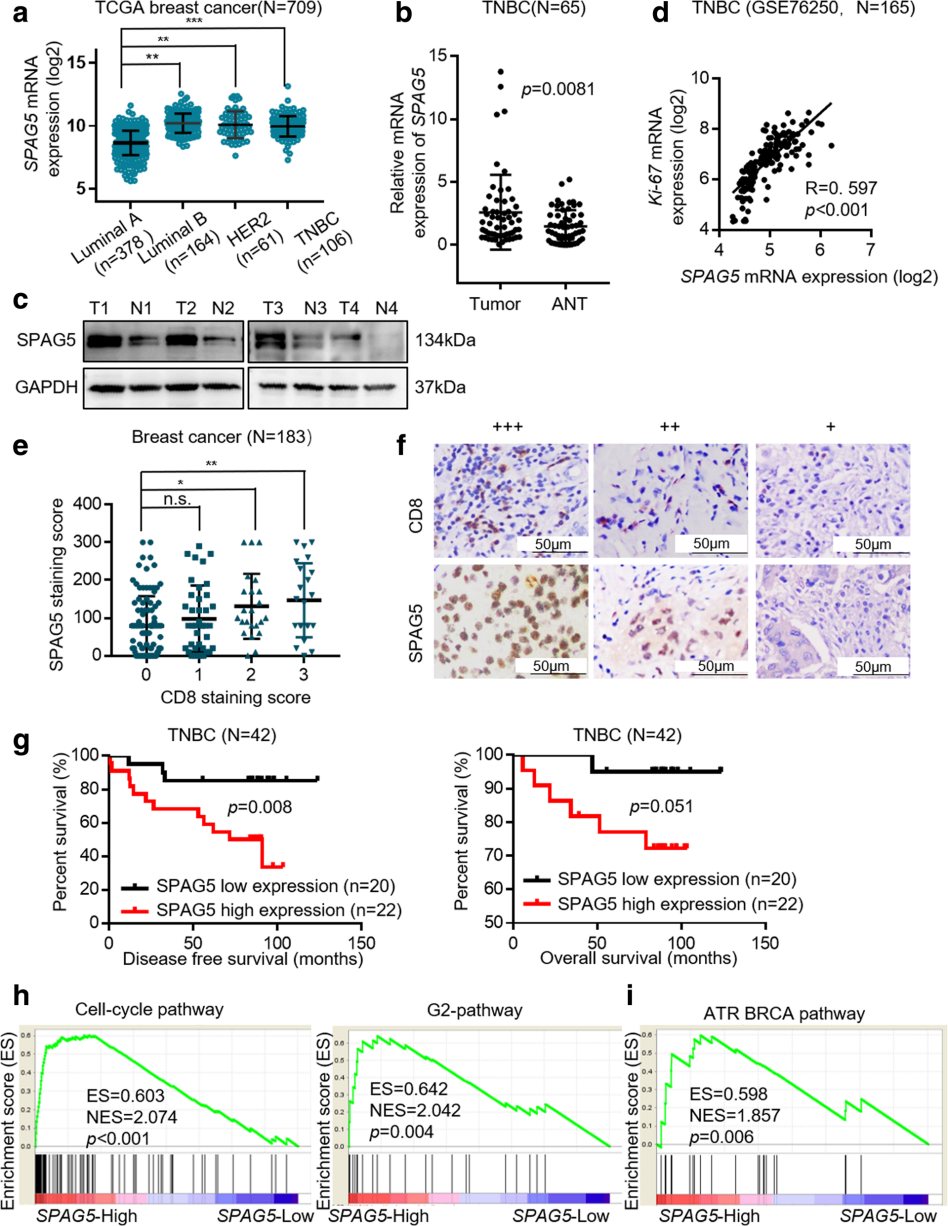
Increased SPAG5 expression promotes TNBC progression and correlates with poor prognosis. **a** SPAG5 mRNA levels in TCGA breast cancer mRNA dataset of different molecular subtypes of breast cancer. **b** SPAG5 mRNA levels in paired TNBC tumor tissues versus non-tumor tissues (*n* = 65).**c** Protein expression of SPAG5 in TNBC cases were examined by western blot. **d** Correlation of SPAG5 and ki-67 mRNA levels in GSE76250 dataset. **e** Correlation of SPAG5 and CD8 protein expression levels. **f** Representative IHC image of SPAG5 expression and CD8 expression in breast cancer specimens. **g** Kaplan–Meier curve of DFS and OS for TNBC patients with low expression of SPAG5 versus high expression of SPAG5 group. **h** Gene expression data acquired from TCGA (the group of SPAG5 mRNA high TNBC and SPAG5 mRNA low TNBC) were subjected to GSEA using GSEA v2.2.0 showed that high SPAG5 expression positively correlated with cell cycle-related signatures and G2 related signatures. **i** The GSEA plot showed that high SPAG5 expression positively correlated with cell ATR BRCA pathway. All *p<0.05, **p<0.01, ***p<0.001, n.s. not significant

SPAG5 mRNA expression was upregulated in tumor compared with that in normal tissues in TCGA breast cancer dataset (*p <* 0.001, Additional file 2: Fig. S1a) and was high in TNBC compared with that in luminal A breast cancer (*p <* 0.001, Fig. 1a). SPAG5 mRNA was significantly upregulated in TNBC tumor tissues compared with that in the paired ANTs in our cohort (*p* = 0.008, Fig. 1b), which is consistent with the findings in the GSE76250 TNBC dataset (*p <* 0.001, Additional file 2: Fig. S1b), and SPAG5 protein was also unregulated (Fig. 1c). In addition, SPAG5 mRNA expression was positively correlated with Ki-67 mRNA expression in 165 TNBC cases from the GSE76250 data (R = 0. 597, *p <* 0.001, Fig. 1d), which indicates that SPAG5 is a proliferation marker in TNBC.

SPAG5 protein expression was examined by IHC in 183 breast cancer samples, including 42 TNBC samples. High SPAG5 expression was associated with more CD8+ T cell infiltration in breast cancer (Fig. 1e, f), which suggested SPAG5 could be a potential candidate for future vaccine development. In breast cancer, we found that high SPAG5 expression was associated with increased local recurrence (*p <* 0.001, Additional file 3: Table S2). SPAG5 upregulation in tumor tissues indicated poor disease-free survival (DFS, HR = 2.470, 95%CI 1.203–5.073, *p* = 0.016) and overall survival (OS, HR = 3.327, 95%CI 1.204–9.196, *p* = 0.029, Additional file 2: Fig. S1c) and it was also an independent prognostic factor for breast cancer patients (Additional file 4: Table S3). Furthermore, we found that high SPAG5 expression was associated with increased lymph node metastasis (*p* = 0.040) and increased risk of local recurrence (*p* = 0.009, Table 1) in TNBC. High SPAG5 expression also indicated poor DFS (HR = 4.639, 95%CI 1.681–12.8, *p* = 0.008, Table 2) in TNBC, but not poor OS (*p* = 0.051) (Fig. 1g and Additional file 5: Table S4). Taken together, upregulated SPAG5 expression is related to poor prognosis in TNBC patients.

**Table 1:** Correlation of SPAG5 expression and clinical features of TNBC patients

Variable	Overall (*N* = 42)		SPAG5				
			Low expression (*N* = 20)		High expression (*N* = 22)		
	*N*	%	*N*	%	*N*	%	*P*
Age, years							0.746
≤ 50	20	47.62	9	45.00	11	50.00	
> 50	22	52.38	11	55.00	11	50.00	
Tumor size, cm							0.72
< 2	21	50.00	9	45.00	12	54.55	
2 ≤ T < 5	18	42.86	9	45.00	9	40.91	
≥ 5	3	7.14	2	10.00	1	4.55	
Histological grade							0.98
I/II	23	54.76	11	55.00	12	54.55	
III	19	45.24	9	45.00	10	45.45	
Node status							*0.04*
pN0 (none)	22	52.38	12	60.00	10	45.45	
pN1 (1–3)	8	19.05	3	15.00	5	22.73	
pN2 (4–9)	4	9.52	4	20.00	0	0.00	
pN3 (≥ 10)	7	16.67	1	5.00	6	27.27	
pNX	1	2.38	0	0.00	1	4.55	
Local recurrence							*0.009*
Absence	35	83.33	20	100.00	15	68.18	
Presence	7	16.67	0	0.00	7	31.82	
Distant metastasis							0.243
Absence	34	80.95	18	90.00	16	72.73	
Presence	8	19.05	2	10.00	6	27.27

**Table 2:** Univariate and multivariate analyses of SPAG5 expression and DFS in TNBC patients

Variable	DFS					
	Univariate analysis			Multivariate analysis		
	HR	95% CI	*P*	HR	95% CI	*P*
SPAG5	4.639	1.681–12.800	*0.008*	4.475	1.328–16.958	*0.017*
Age	1.465	0.521–4.122	0.469			
Tumor size	0.984	0.415–2.334	0.98			
Histological grade	0.964	0.380–2.443	0.939			
Node status	1.599	0.576–4.440	0.368			

To further explore the potential functions of SPAG5 in TNBC, we performed a gene set enrichment analysis (GSEA) using mRNA expression data from TCGA database, and the results showed that high SPAG5 expression was significantly correlated with cell-cycle-related genes and G2-phase-related genes (Fig. 1h), including CDC25C, CDC20, CCNE1, E2F1, and E2F2. Interestingly, high SPAG5 expression also correlated with ATR-BRCA pathway-related genes (Fig. 1i), including BRCA1, BRCA2, RAD51, and EXO1.

### SPAG5 promotes TNBC cell proliferation in vitro and in vivo

**Fig. 2:**
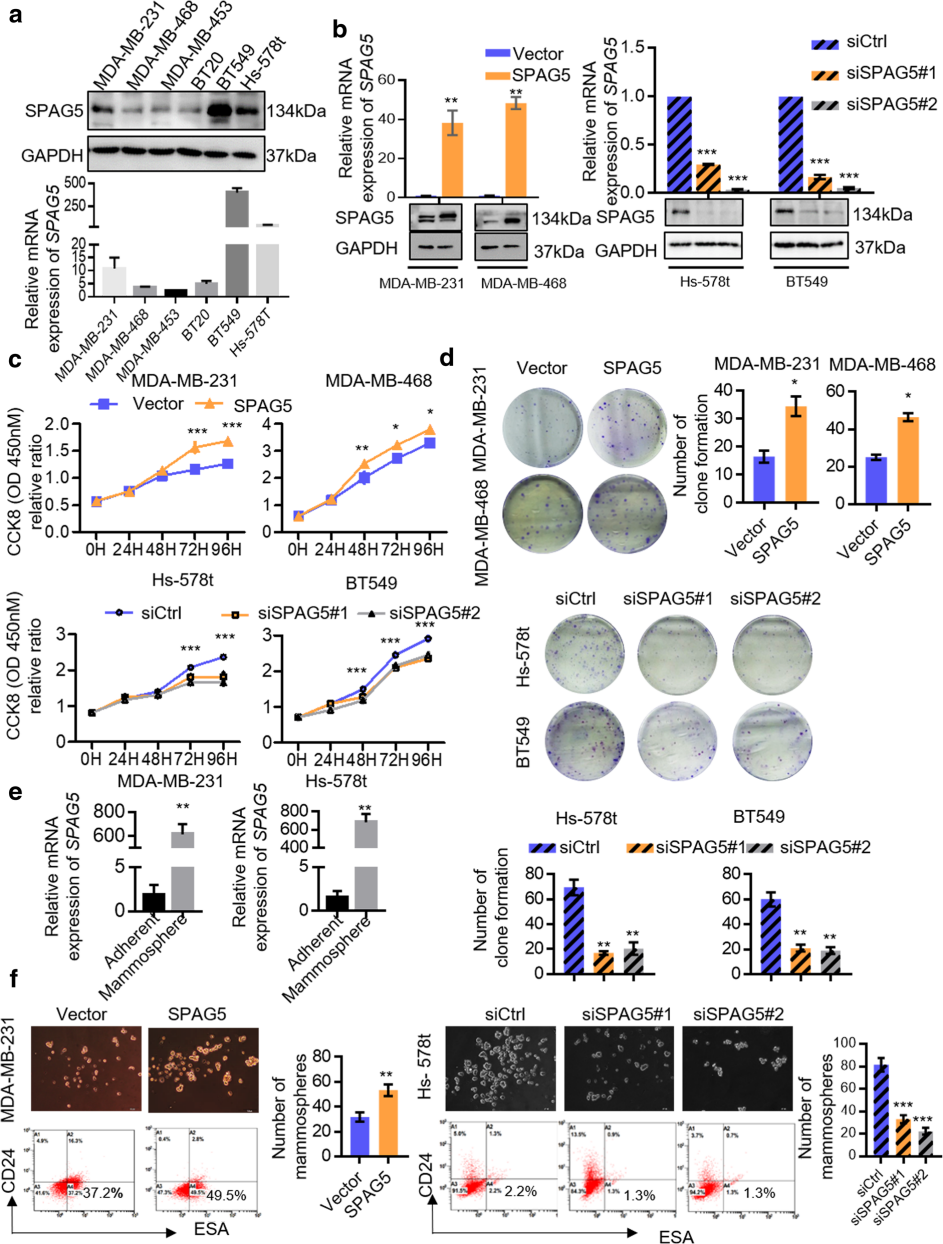
SPAG5 expression promotes the proliferation of TNBC in vitro. **a** mRNA and protein expression levels of SPAG5 in TNBC cell lines. **b** Western blot and RT-qPCR results show the efficiencies of SPAG5 overexpression in MDA-MB-231 and MDA-MB-468 cells and SPAG5 knockdown in Hs-578t and BT549 cells. CCK-8 assays (**c**) and colony-forming (**d**) assays showed that overexpression of SPAG5 promoted cell proliferation in MDA-MB-231 and MDA-MB-468 cells, while knockdown of SAPG5 suppressed cell proliferation in Hs-578t and BT549 cells. **e** Total RNA was isolated from MDA-MB-231 and Hs-578t cells grown as adherent cultures, mammospheres and assayed for SPAG5 expression by qRT-PCR. **f** MDA-MB-231 and Hs-578t cells were plated on low adhesion plates to measure their ability to form mammospheres. Flow-cytometric analysis of CD24 − ESA+ population in MDA-MB-231 and Hs-578t cells grown as adherent culture. All *p<0.05, **p<0.01, ***p<0.001, n.s. not significant

To investigate the potential effect of SPAG5 in TNBC, we first determined SPAG5 expression levels in six TNBC cell lines (Fig. 2a). MDA-MB-231 and MDA-MB-468 cells with low basal SPAG5 expression were selected for overexpression, while Hs-578t and BT549 cells with relatively high basal SPAG5 expression were selected for knockdown. Western blotting and qRT-PCR analyses were used to confirm the efficiencies of overexpression and knockdown (Fig. 2b). CCK8 (Fig. 2c) and colony formation (Fig. 2d) assays revealed that overexpression of SPAG5 in MDA-MB-231 and MDA-MB-468 cells significantly promoted cell proliferation, while SPAG5 knockdown in Hs-578t and BT549 cells suppressed cell proliferation. Moreover, SPAG5 expression was significantly elevated in the mammospheres generated from MDA-MB-231 and Hs-578t cells compared with that in adherent cells (Fig. 2e). Overexpression of SPAG5 in MDA-MB-231 cells increased the sphere-forming ability and the population of CD24-ESA+ cancer stem cells (CSCs), while SPAG5 knockdown had the opposite effects on Hs-578t cells (Fig. 2f).

**Fig. 3:**
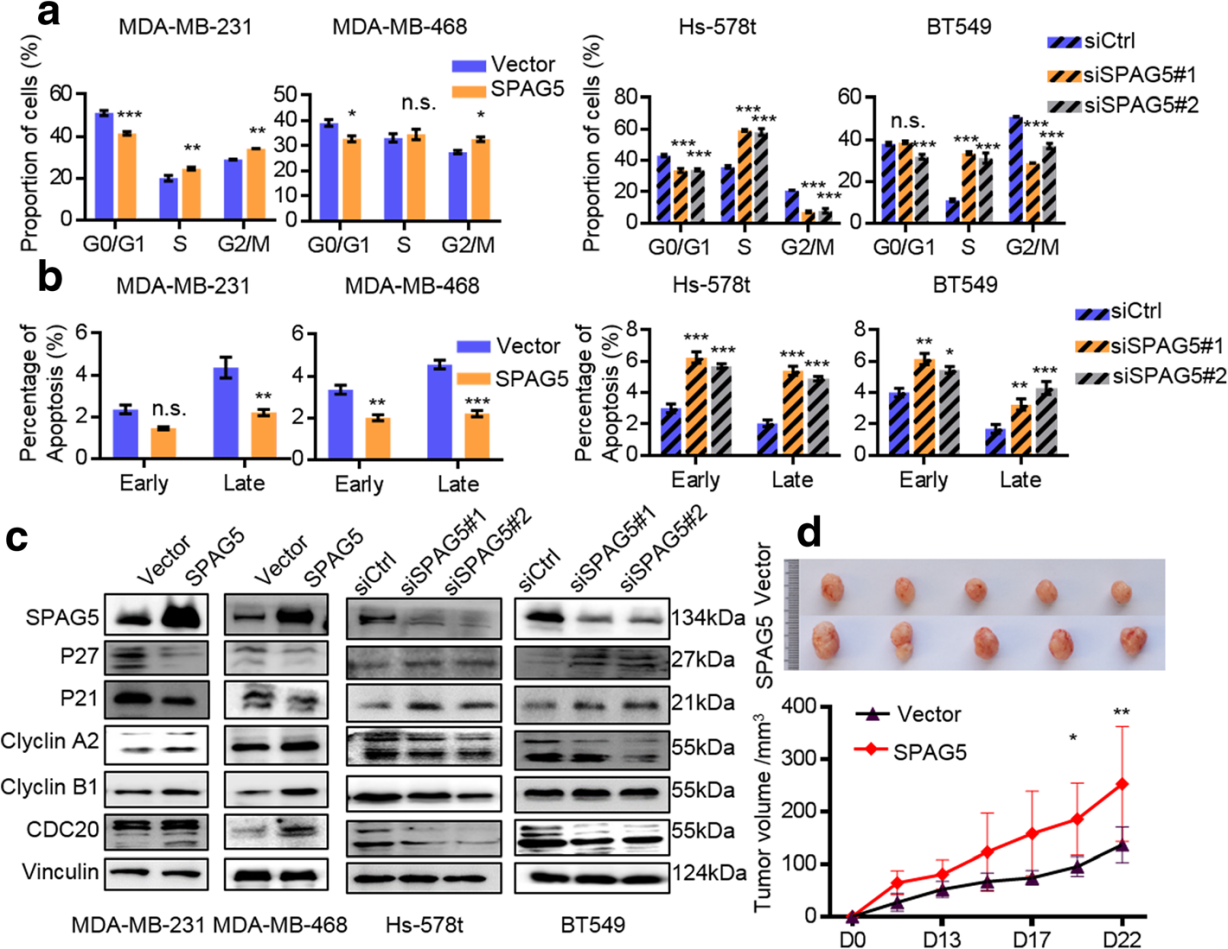
SPAG5 expression promotes cell cycle progression and inhibits cell apoptosis of TNBC. **a** Flow cytometry indicates that overexpression of SPAG5 promotes S to G2 transition in MDA-MB-231 and MDA-MB-468 cells, while knockdown of SPAG5 suppress S to G2 transition in Hs-578t and BT549 cells. **b** Flow cytometry indicates that overexpression of SPAG5 inhibit cell apoptosis in MDA-MB-231 and MDA-MB-468 cells, while knockdown of SPAG5 increase cell apoptosis in Hs-578t and BT549 cells. **c** Western blot of SPAG5, P21, P27, Cyclin A2, Cyclin B1, and CDC20 in MDA-MB-231 and MDA-MB-468 cells treated with SPAG5 overexpression and Hs-578t and BT549 cells treated with SPAG5 siRNAs. **d** Tumors formed by SPAG5 overexpression MDA-MB-231 cells were larger than those formed by cells transduced with the vector control. All *p<0.05, **p<0.01, ***p<0.001, n.s. not significant

Next, we examined the effect of SPAG5 on cell-cycle progression and cell apoptosis by flow cytometry. Overexpression of SPAG5 increased the G2-phase cell population and decreased the G1-phase cell population, whereas SPAG5 knockdown induced S to G2 arrest, indicating that SPAG5 promotes the S/G2 transition (Fig. 3a and Additional file 6: Fig. S2a). Regarding to apoptosis, ectopic SPAG5 expression inhibited apoptosis in MDA-MB-231 and MDA-MB-468 cells, while SPAG5 knockdown induced apoptosis in Hs-578t and BT549 cells (Fig. 3b and Additional file 6: Fig. S2b). Western blotting revealed that key mediators of the cell cycle and apoptosis, namely, Cyclin A2, Cyclin B1, and CDC20, were positively regulated by SPAG5, while P21 and P27 were negatively regulated (Fig. 3c).

To assess the effect of SPAG5 on tumorigenesis in vivo, we generated xenografts through subcutaneous injection of MDA-MB-231 cells stably expressing SPAG5 or vector and found that the volumes of the tumors formed by the SPAG5 overexpressing MDA-MB-231 cells were significantly greater than those formed by the control cells (Fig. 3d). Taken together, these results suggest that SPAG5 plays an important tumorigenic role in promoting TNBC cell growth in vitro and in vivo.

### SPAG5 knockdown results in enhanced tumor sensitivity to olaparib

Because SPAG5 expression is significantly associated with the ATR BRCA pathway-related genes RAD51, BRCA1, and BRCA2 and due to its essential role in cell cycle progression, we assumed that SPAG5 may affect DNA repair pathways and PARPi activity.

**Fig. 4:**
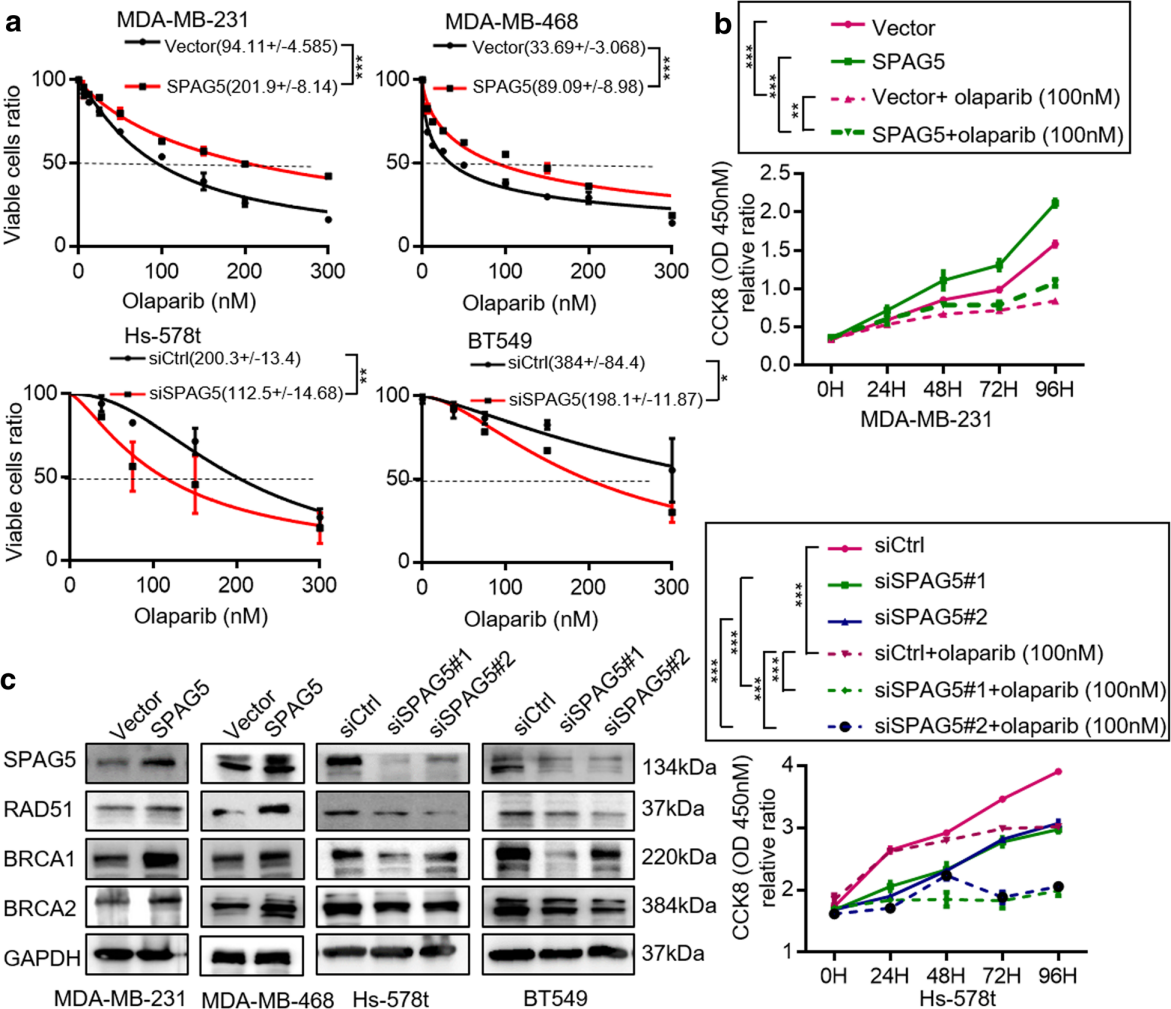
SPAG5 knockdown results in enhanced sensitivity to olaparib. **a** CCK8 assays showed IC50 values for olaparib in SPAG5 overexpression MDA-MB-231 and MDA-MB-468 cells and SPAG5 knockdown Hs-578t and BT549 cells. **b** CCK-8 assays showed overexpression of SPAG5 reduced the efficiency of olaparib in MDA-MB-231 cells, while knockdown of SPAG5 increased the efficiency of olaparib in Hs-578t cells. **c** Western blot of SPAG5, RAD51, and BRCA2 in MDA-MB-231and MDA-MB-468 cells treated with SPAG5 overexpression and Hs-578t and BT549 cells treated with SPAG5 siRNAs

SPAG5 knockdown Hs-578t and BT549 cells were significantly more sensitive to the PARPi olaparib than the negative control cells (Fig. 4a). Consistently, SPAG5 overexpressing MDA-MB-231 and MDA-MB-468 cells were more resistant to olaparib than the vector control cells (Fig. 4a). CCK8 assays indicated that SPAG5 knockdown promoted the efficacy of olaparib in Hs-578t cells, while SPAG5 overexpression decreased the efficacy of olaparib in MDA-MB-231 cells (Fig. 4b). Moreover, western blots showed that RAD51, BRCA1, and BRCA2 expression were positively associated with SPAG5 expression (Fig. 4c). These data suggest that SPAG5 plays an important role in DNA repair, which leads to decreased sensitivity to the PARPi olaparib in TNBC.

### SPAG5 upregulates MYCBP expression and transcription of c-MYC target genes through direct interaction with the MYCBP protein

MYCBP has been shown to interact with SPAG5 and form the Astrin-SKAP complex, and it plays a critical role in chromosome segregation in human cells. MYCBP is a c-MYC binding protein that stimulates the transcriptional activity of c-MYC; thus, we further examined whether MYCBP/c-MYC plays a role in the molecular regulation by which SPAG5 alters cell cycle progression and DNA repair.

**Fig. 5:**
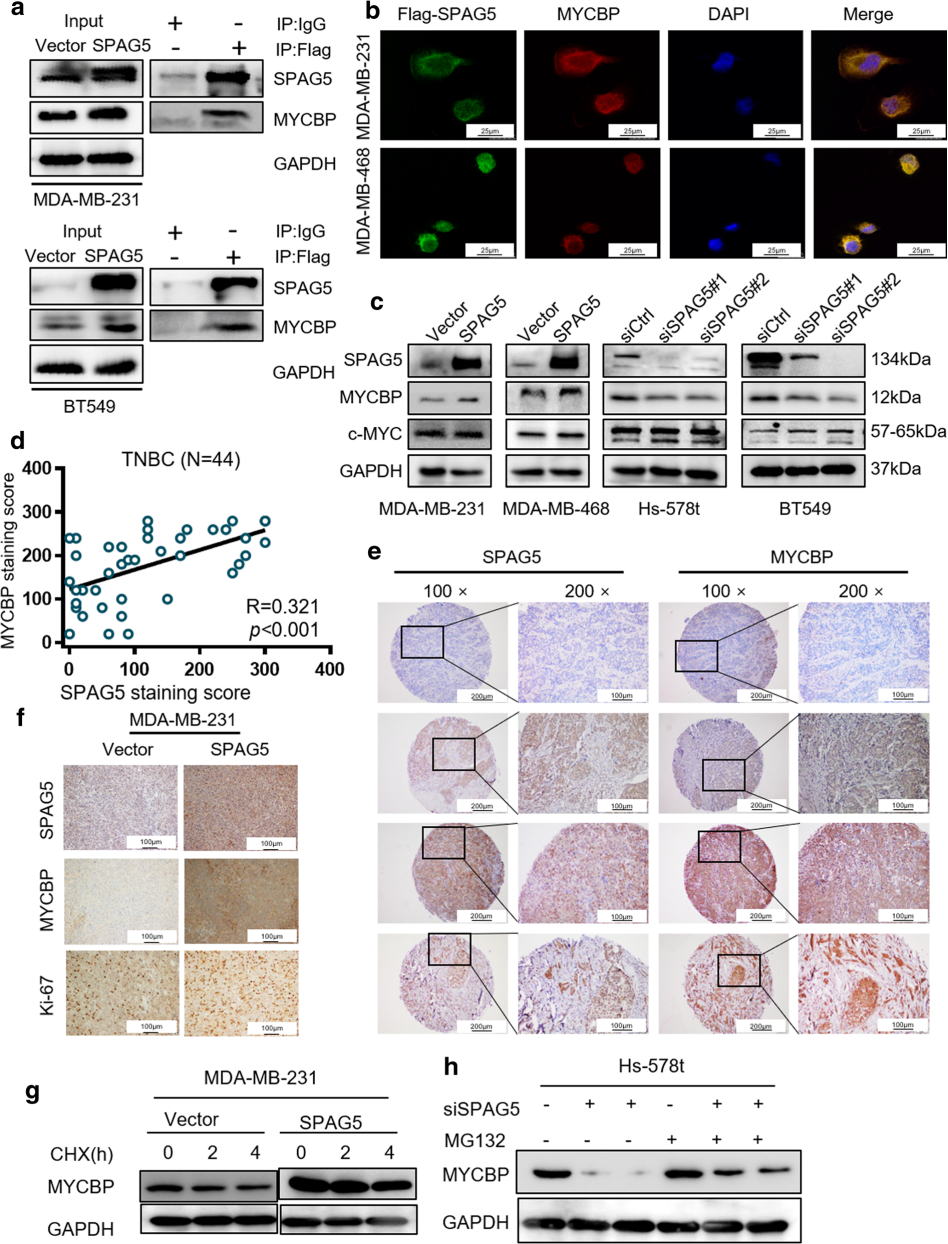
SPAG5 upregulates MYCBP expression through direct interaction with MYCBP protein. **a** IP assays suggested that exogenously expressed Flag-SPAG5 specifically interacted with endogenous MYCBP in MDA-MB-231 and BT549 cells. **b** IF staining experiments showed that SPAG5 mainly localizes in the cytoplasm with MYCBP in MDA-MB-231 and MDA-MB-468 cells. **c** Western blot results suggested that the protein level of MYCBP was elevated in SPAG5- overexpressing MDA-MB-231 and MDA-MB-468 cells but reduced in SPAG5-knockdown Hs-578t and BT549 cells. **d** Regression analysis identified a positive relationship between SPAG5 and MYCBP protein expression levels in TNBC tissues. **e** Representative IHC image of SPAG5 expression and MYCBP expression in breast cancer specimens. **f** Representative IHC image of SPAG5 expression, MYCBP expression, and Ki-67 staining in xenografted MDA-MB-231 tumor specimens. **g** MDA-MB-231 cells were transfected with p-SPAG5 plasmid and treated with cycloheximide (CHX). Cells were collected at different time points and immunoblotted with MYCBP antibody. **h** Hs-578t cells transduced with SPAG5 siRNA were treated with 10 μM MG132 and collected at 6 h and immunoblotted with the MYCBP antibody

First, we validated the interaction of MYCBP and SPAG5 in TNBC cells. Due to the lack of available SPAG5 antibodies for IP analysis, MDA-MB-231 and BT549 cells were transfected with Flag-SPAG5. IP analysis with an anti-Flag antibody showed that exogenously expressed Flag-SPAG5 specifically interacted with endogenous MYCBP (Fig. 5a). Consistently, immunofluorescence (IF) staining experiments showed that SPAG5 mainly localizes in the cytoplasm and co-localizes with endogenous MYCBP in MDA-MB-231 and BT549 cells (Fig. 5b). SPAG5 expression positively regulated MYCBP protein expression in TNBC cells (Fig. 5c) without affecting the mRNA levels of MYCBP and c-MYC protein and mRNA expression (Additional file 7: Fig. S3a). Then, we examined MYCBP expression in 183 breast cancer patients using IHC and found that MYCBP protein expression is positively correlated with SPAG5 expression in breast cancer (R = 0.218, *p <* 0.001, Additional file 7: Fig. S3b), as well as in TNBC (R = 0.321, *p <* 0.001, Fig. 5d, e). Importantly, IHC staining in tumors generated from xenografts showed that high SPAG5 expression displayed high MYCBP and Ki67 staining (Fig. 5f). These data confirmed that SPAG5 interacts with MYCBP and increases MYCBP protein expression in TNBC. To further investigate whether SPAG5 regulates the stability of MYCBP protein, we evaluated MYCBP protein levels in the presence of cycloheximide (CHX), an inhibitor of translation. Notably, overexpression of SPAG5 in MDA-MB-231 cells led to a significant increase in MYCBP protein stability (Fig. 5g). We further assessed the mechanisms through which SPAG5 regulates MYCBP. Our results showed that the expression of MYCBP protein was increased after dysregulation of SPAG5 in the presence of the proteasome inhibitor MG132 (Fig. 5h). Therefore, these results confirmed that SPAG5 increase MYCBP protein expression through modifying MYCBP protein stability.

**Fig. 6:**
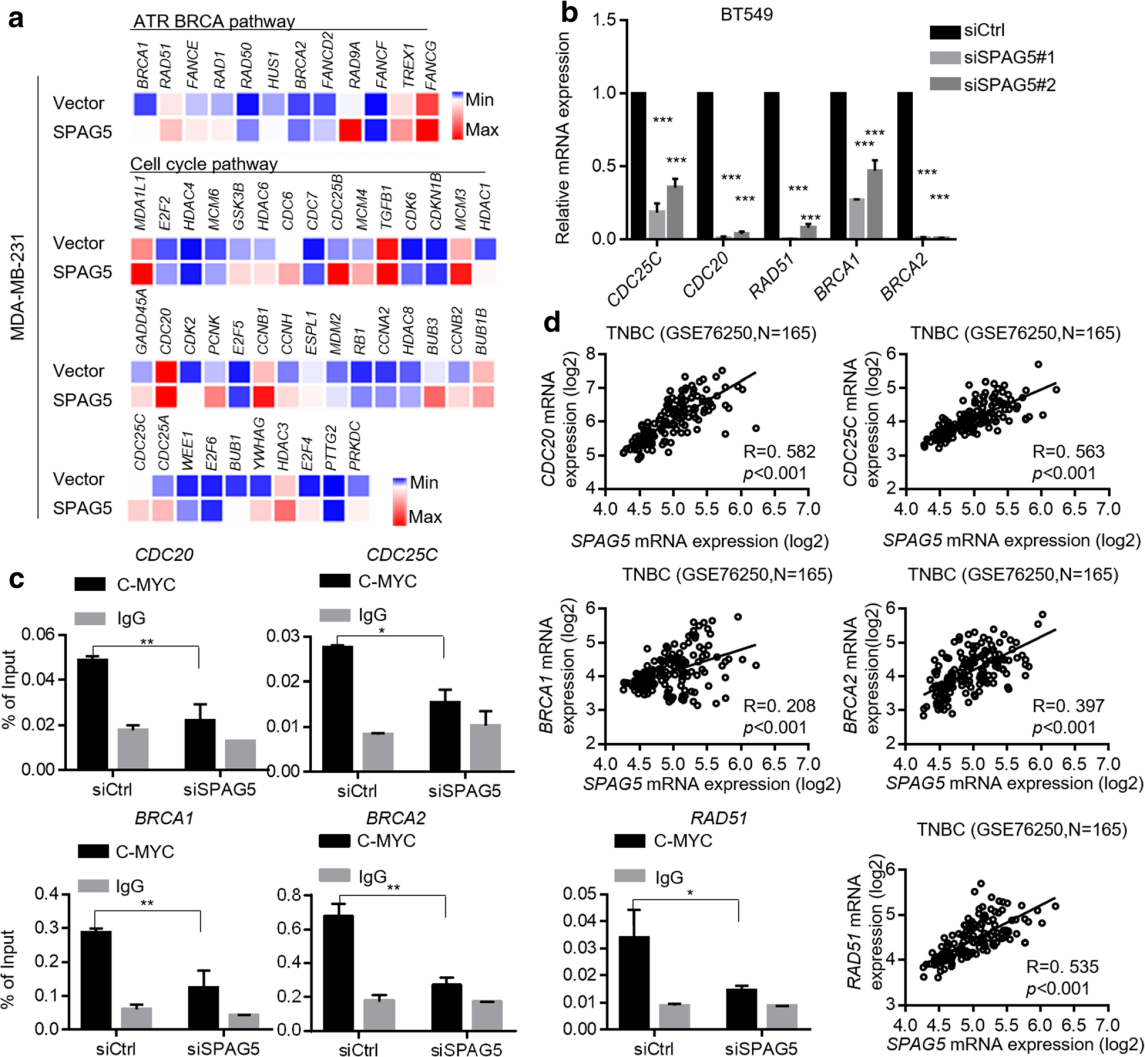
SPAG5 upregulates transcription of c-MYC target genes by regulating c-MYC transcriptional activity. **a** Cell cycle-related genes and ATR BRCA pathway related genes showed differential mRNA expression in SPAG5 overexpression cells compared with that in vector cells in RNA-sequencing analysis. **b** RT-qPCR of CDC20, CDC25C, RAD51, BRCA1, and BRCA2 in BT549 cells treated with SPAG5 siRNAs. **c** Correlation of SPAG5 and CDC20, CDC25C, RAD51, BRCA1, and BRCA2 mRNA levels in GSE76250 dataset. **d** CHIP-qPCR showed SPAG5 knockdown in BT549 cells significantly reduced c-MYC’s occupancies on the promoters of CDC20, CDC25C, RAD51, BRCA1, and BRCA2. All *p<0.05, **p<0.01, ***p<0.001, n.s. not significant

Furthermore, RNA-sequencing was used to compare the global gene expression profiles of SPAG5 overexpressing MDA-MB-231 cells with the vector control cells (Additional file 8: Table S5). Consistent with the GSEA results, upregulation of cell-cycle-related and ATR-BRCA pathway-related genes was confirmed in SPAG5 overexpressing MDA-MB-231 cells compared with their expression in the vector control cells (Fig. 6a). qRT-PCR was used to validate mRNA expression of the c-MYC targets CDC25C, CDC20, RAD51, BRCA1, and BRCA2. They were all significantly upregulated by SPAG5 overexpression and downregulated by SPAG5 knockdown in TNBC cells (Fig. 6b and Additional file 7: Fig. S3d). It was also confirmed that mRNA expression of the c-MYC targets CDC25C, CDC20, RAD51, BRCA1, and BRCA2 was significantly downregulated by c-MYC knockdown in MDA-MB-231 and MDA-MB-231 cells (Additional file 7: Fig. S3e). CHIP-qPCR was performed to investigate whether SPAG5 affected gene expression by regulating c-MYC transcriptional activity (Additional file 7: Fig. S3c). As shown by qPCR, SPAG5 knockdown in BT549 cells significantly reduced the occupancy of c-MYC on the promoters of CDC20, CDC25C, RAD51, BRCA1, and BRCA2 (Fig. 6c). Correlations between SPAG5 expression and these c-MYC targets in 165 TNBC patients from the GSE76250 data were all significantly positive (Fig. 6d). Collectively, we demonstrate that SPAG5 could interact with MYCBP and increase MYCBP expression, which resulted in increased c-MYC transcriptional activity in TNBC.

### SPAG5 facilitates TNBC tumor proliferation in a MYCBP/c-MYC-mediated manner

**Fig. 7:**
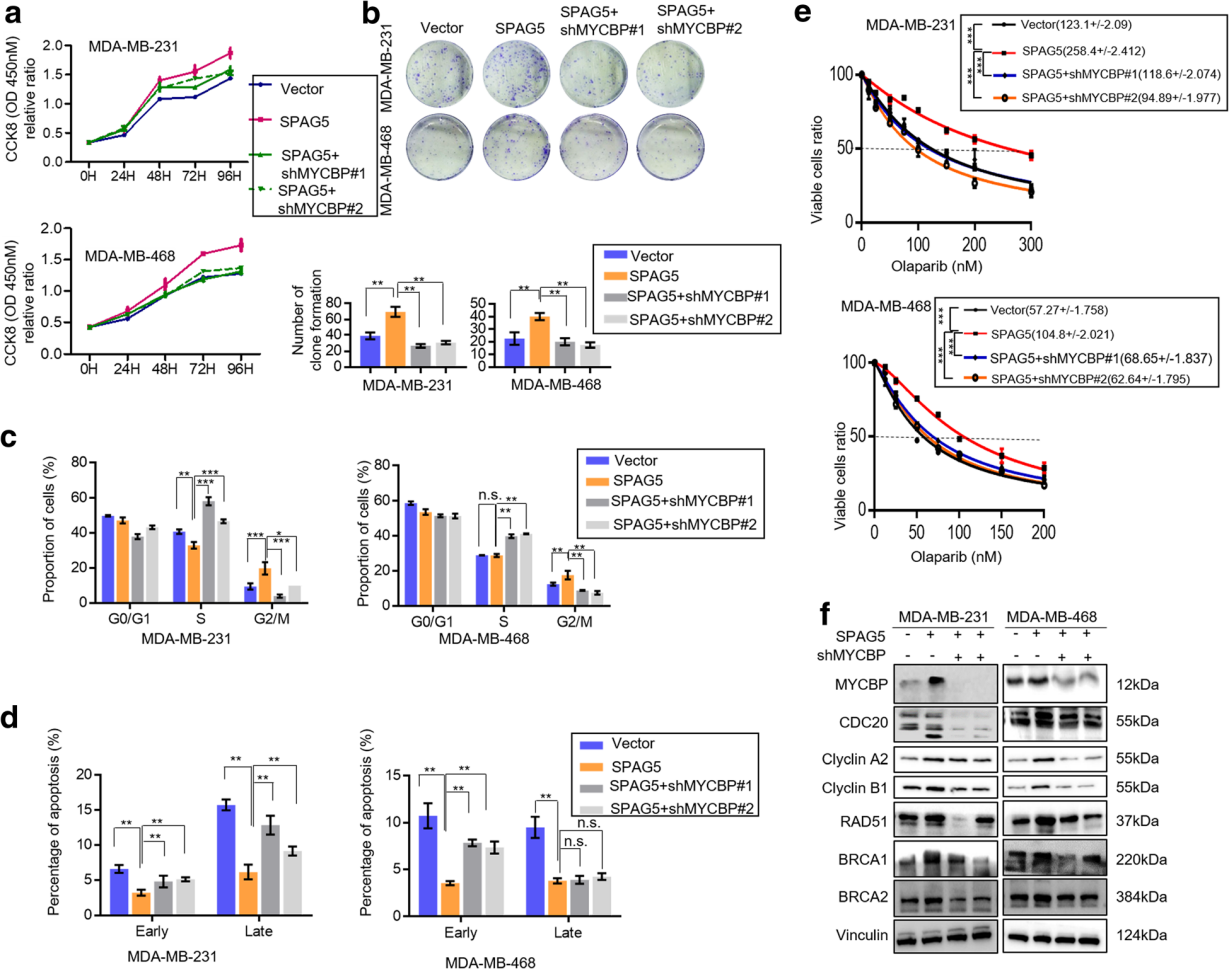
MYCBP is partially involved in SPAG5 regulated breast cancer cell growth. CCK-8 (**a**) and colony-forming (**b)** assays showed that knockdown of MYCBP partially attenuated the enhanced cell proliferation induced by overexpression of SPAG5 in MDA-MB-231 and MDA-MB-468 cells. **c** Flow cytometry indicates that knockdown of MYCBP partially reverse the increased S to G2 transition induced by overexpression of SPAG5 in MDA-MB-231 and MDA-MB-468 cells. **d** Flow cytometry indicates that knockdown of MYCBP partially reverse the reduced cell apoptosis induced by overexpression of SPAG5 in MDA-MB-231 and MDA-MB-468 cells. **e** CCK8 assays showed IC50 values for olaparib in SPAG5 overexpression MDA-MB-231 and MDA-MB-468 cells transfected with MYCBP shRNAs. **f** MDA-MB-231and MDA-MB-468 cells stably expressing SPAG5 were infected with shRNAs targeting MYCBP. Western blot was used to detect MYCBP, CDC20, Cyclin A2, Cyclin B1, RAD51, BRCA1, and BRCA2 protein expression level. All *p<0.05, **p<0.01, ***p<0.001, n.s. not significant

The function of MYCBP on TNBC was first determined in Hs-578t cells (Additional file 9: Fig. S4). To further elucidate whether SPAG5 regulated TNBC cell-cycle progression and sensitivity to olaparib by increasing MYCBP expression, we transfected sh-MYCBP plasmids into MDA-MB-231 and MDA-MB-468 cells with stabled SPAG5 overexpression. We found that knockdown of MYCBP significantly abolished the positive effects of SPAG5 on cell proliferation (Fig. 7a, b). Cell cycle analysis indicated that knockdown of MYCBP significantly abolished the positive effects of SPAG5 on cell-cycle progression (Fig. 7c and Additional file 10: Fig. S5a). Apoptosis assay indicated that MYCBP knockdown reversed the inhibitory effects of SPAG5 on cell apoptosis (Fig. 7d and Additional file 10: Fig. S5b). The resistance to olaparib induced by SPAG5 overexpressing in MDA-MB-231 and MDA-MB-468 cells was also significantly abolished by MYCBP knockdown (Fig. 7e). Furthermore, the increased expression of CDC20, Cyclin A2, Cyclin B1, RAD51, BRCA1, and BRCA2 induced by SPAG5 was partly blocked by incubation with MYCBP shRNAs (Fig. 7f).

**Fig. 8:**
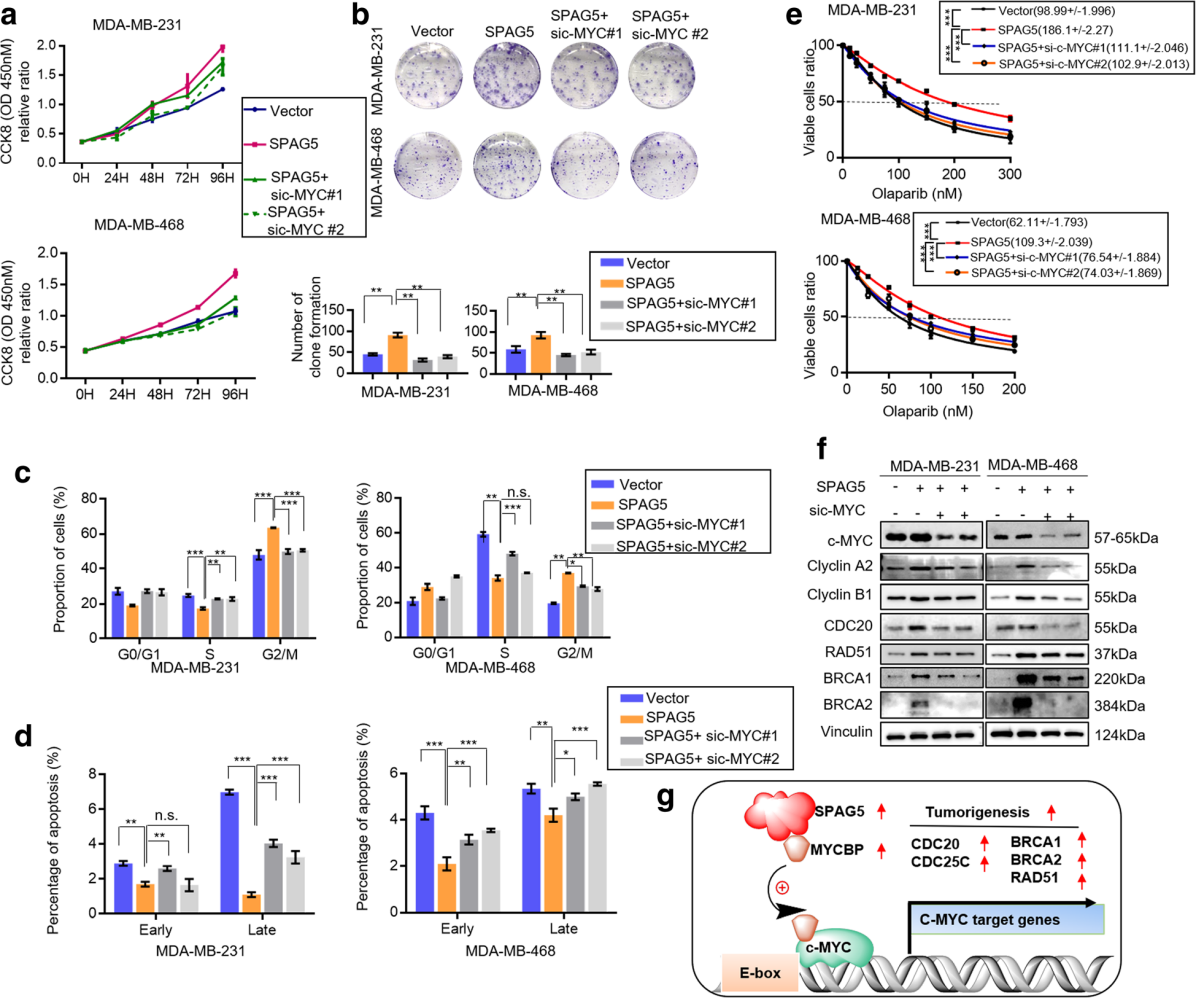
c-MYC is partially involved in SPAG5 regulated breast cancer cell growth. CCK-8 (**a**) and colony-forming (**b**) assays showed that knockdown of c-MYC partially attenuated the enhanced cell proliferation induced by overexpression of SPAG5 in MDA-MB-231 and MDA-MB-468 cells. **c** Flow cytometry indicates that knockdown of c-MYC partially reverse the increased S to G2 transition induced by overexpression of SPAG5 in MDA-MB-231 and MDA-MB-468 cells. **d** Flow cytometry indicates that knockdown of c-MYC partially reverse the reduced cell apoptosis induced by overexpression of SPAG5 in MDA-MB-231 and MDA-MB-468 cells. **e** CCK8 assays showed IC50 values for olaparib in SPAG5 overexpression MDA-MB-231 and MDA-MB-468 cells transfected with c-MYC siRNAs. **f** MDA-MB-231and MDA-MB-468 cells stably expressing SPAG5 were infected with siRNAs targeting c-MYC. Western blot was used to detect c-MYC, CDC20, Cyclin A2, Cyclin B1, RAD51, BRCA1 and BRCA2 protein expression level. **g** Model showing the effect of SPAG5/MYCBP/c-MYC signaling on breast tumor growth. All *p<0.05, **p<0.01, ***p<0.001, n.s. not significant

Then, we investigated whether SPAG5 regulated TNBC cell-cycle progression by increasing c-MYC transcriptional activity and we transfected siRNAs of c-MYC into MDA-MB-231 and MDA-MB-468 cells with stabled SPAG5 overexpression. We found that knockdown of c-MYC significantly abolished the positive effects of SPAG5 on cell proliferation (Fig. 8a, b). Cell cycle analysis indicated that knockdown of c-MYC significantly abolished the positive effects of SPAG5 on cell-cycle progression (Fig. 7c and Additional file 9: Fig. S5a). Apoptosis assay indicated that c-MYC knockdown reversed the inhibitory effects of SPAG5 on cell apoptosis (Fig. 8d and Additional file 9: Fig. S5b). The resistance to olaparib induced by SPAG5 overexpressing in MDA-MB-231 and MDA-MB-468 cells was also significantly abolished by MYCBP knockdown (Fig. 8e). Furthermore, the increased expression of CDC20, Cyclin A2, Cyclin B1, RAD51, BRCA1, and BRCA2 induced by SPAG5 was partly blocked by incubation with c-MYC siRNAs (Fig. 8f).

These results provide evidence that the oncogenic role of SPAG5 is mediated through the regulation of MYCBP expression and c-MYC transcriptional activity.

## Discussion

In this study, we found that SPAG5 expression was upregulated in TNBC, which correlated with poor DFS. Furthermore, we demonstrated that SPAG5 promoted tumor growth by increasing c-MYC transcriptional activity via interaction with MYCBP (Fig. 8g). In addition, SPAG5 knockdown increased the sensitivity to the PARPi olaparib in TNBC cells.

Notably, there is an established body of research in several types of cancer showing that SPAG5 is involved in tumorigenesis and patient prognosis. Increased SPAG5 expression was reported to be accompanied by reduced survival in hepatocellular carcinoma [21]. SPAG5 upregulation was related to poor prognosis in cervical cancer patients [9]. SPAG5 expression in prostate cancer was associated with cancer progression and unfavorable outcomes [10]. For breast cancer, the analysis of multiple breast cancer cohorts indicated that high SPAG5 transcript expression was associated with decreased survival in all breast cancer subtypes and estrogen receptor-positive subgroups; although, its prognostic value in TNBC was not clear [7]. Accordingly, our data suggested that high SPAG5 expression is associated with worse patient outcomes, which are consistent with the previous study. Moreover, SPAG5 was upregulated in TNBC and correlated with worse DFS.

The investigation of the biological function of SPAG5 in TNBC suggested that SPAG5 prevented TNBC cells from undergoing apoptosis, which is consistent with a previous study in MCF7 cells [22]. The GSEA analysis of TNBC suggested that high SPAG5 expression is associated with cell-cycle-related genes (CDC20 and CDC25C) and ATR BRCA pathway-related genes (RAD51, BRCA2, BRCA1). It has been reported that ATR enforces an S/G2 transition to promote genome integrity [23]. The cell cycle analysis in our study also indicated that SPAG5 could promote the S/G2 transition. Ectopic SPAG5 expression increased Cyclin B1, Cyclin A2, and CDC20 expression, but decreased P21, P27 expression. In vitro and in vivo assays were used to investigate the role of SPAG5 in regulation of TNBC cell proliferation. The results showed that SPAG5 overexpression promoted TNBC cells proliferation. All data suggested that SPAG5is involved in tumor growth of TNBC.

Our study also demonstrated that SPAG5 expression levels are related to sensitivity to olaparib of TNBC. Olaparib is a PARPi that has been used in clinical trials targeting breast or ovarian cancers. Our findings showed that SPAG5 overexpression decreased the sensitivity of MDA-MB-231 and MDA-MB-468 cells to olaparib, while SPAG5 knockdown increased the sensitivity to olaparib. SPAG5 overexpression increased RAD51, BRCA1, and BRCA2 mRNA and protein expression, while SPAG5 knockdown reduced their expression. Moreover, SPAG5 mRNA expression was positively correlated with RAD51, BRCA1, and BRCA2 expression in TNBC. In current clinical management, BRCA1/BRCA2 mutations remain the most frequently used biomarkers of HR DNA repair deficiency in TNBC to determine whether patients are suitable for PARPi treatment or not. However, whether PARPi has potential effects in addition to BRCA1/BRCA2 mutation carriers remains an important question. Evidence has suggested that MYC expression can be used to stratify TNBC patients for therapy with PARPi independent of BRCA status because MYC is a transcriptional regulator of RAD51 [17] The recruitment of the repair protein RAD51 by BRCA2 and BRCA1 to the damaged DNA sites is an important step in HR repair following DNA damage [24]. Recently, a study in ovarian cancer also suggested that BRCA1/2 mRNA levels may be reliable biomarkers to predict responsiveness to PARPi [25]. It was also suggested that cytotoxic PARPi-provoked DNA lesions arise in the context of DNA replications because DNA damage signal primarily occurs in S-phase cells [17]. Thus, the increased S-phase cell population and decreased RAD51 and BRCA1/2 mRNA and protein expression by SPAG5 knockdown is a potential mechanism that explains how reduced SPAG5 expression increases sensitivity to olaparib. Taken together, these data suggest that SPAG5 expression may be used to stratify TNBC patients for PARPi therapy to enable more effective tailoring of PARPi treatment.

Further investigations about the molecular mechanisms of SPAG5 in TNBC confirmed the interaction between SPAG5 and MYCBP [14], a critical c-MYC binding protein that stimulates the activation of E-box regulated transcription by MYC/Max [15]. Reports have indicated that MYCBP plays a key role in cancer growth and progression via c-MYC regulation in breast cancer and other cancer types [26–28]. We observed that SPAG5 knockdown reduced MYCBP protein but not mRNA expression. The IP and IF analyses validated the interaction of SPAG5 and MYCBP. Furthermore, a significant positive correlation between SPAG5 and MYCBP protein expression was also observed in breast cancer and TNBC tissues in our cohort. Further studies are required to elucidate how SPAG5 affect MYCBP protein level. c-MYC has been shown to play critical roles in multiple cellular pathways that promote breast cancer growth and progression [16, 29, 30]. As shown in our results, knockdown of SPAG5 could lead to decreased expression of c-MYC target genes [31–33] related to cell cycle progression and the ATR BRCA pathway. The CHIP-qPCR assay confirmed that SPAG5 knockdown significantly reduces the occupancy of c-MYC on the promoters of CDC20, CDC25C, BRCA1, BRCA2, and RAD51, which supports our speculation that SPAG5 can positively regulate c-MYC transcriptional activity by increasing MYCBP protein expression. To determine whether MYCBP/c-MYC axis is a downstream target involved in SPAG5-induced TNBC growth and apoptosis suppression, we silenced MYCBP and c-MYC in SPAG5 overexpressing cells. The SPAG5-mediated promotion of cell growth and suppression of apoptosis was inhibited dramatically when MYCBP or c-MYC was knocked down. These data suggest that MYCBP is a crucial downstream target of SPAG5 that mediates SPAG5-induced cell growth and SPAG5-suppressed cell apoptosis in TNBC cells.

## Conclusions

In summary, our study indicated that SPAG5 was upregulated in TNBC tissues and that high SPAG5 expression was associated with high risk of local recurrence and poorer outcomes in TNBC patients. SPAG5 promoted TNBC cells growth in vivo and in vitro by enhancing cell cycle progression and suppressing cell apoptosis. Moreover, SPAG5 knockdown increased TNBC cell sensitivity to the PARPi olaparib. Furthermore, SPAG5 interacts with MYCBP, which leads to increased MYCBP protein expression and c-MYC transcriptional activity. It is important to further elucidate the interaction of SPAG5 and MYCBP and test the combination of SPAG5 inhibition and PARPi to achieve an optimal effect in TNBC. Overall, our data suggested that SPAG5 functioned as an oncogenic gene in TNBC by interacting with MYCBP and increasing c-MYC transcriptional activity. Thus, SPAG5 may represent a potential therapeutic target for the clinical intervention of TNBC in the future.

## Additional files

Additional file 1: **Table S1**. Primers for real-time PCR and oligos for siRNAs or shRNAs. (XLSX 11 kb)

Additional file 2: **Figure S1.** Increased SPAG5 expression promotes breast cancer progression and correlates with poor prognosis. a SPAG5 mRNA levels in TCGA breast cancer mRNA dataset of tumor(*n* = 737) versus non-tumor tissues(*n* = 120). b SPAG5 mRNA levels in TNBC (*n* = 165) versus non-tumor tissues(*n* = 33) from GSE76250 dataset. c Kaplan–Meier curve of DFS and OS for breast cancer patients with low expression of SPAG5 versus high expression of SPAG5 group. (PDF 267 kb)

Additional file 3: **Table S2.** Correlation between SPAG5 expression and clinical features of breast cancer patients. (DOCX 35 kb)

Additional file 4: **Table S3.** Univariate and multivariate analyses of SPAG5 expression and prognosis in breast cancer patients. (DOCX 24 kb)

Additional file 5: **Table S4.** Univariate and multivariate analyses of SPAG5 expression and OS in TNBC patients. (DOCX 19 kb)

Additional file 6: **Figure S2.** Representative images of flow cytometry cell cycle analysis(a) and apoptosis analysis(b) in MDA-MB-231, MDA-MB-468 cells, Hs-578t and BT549 cells. (PDF 267 kb) (PDF 148 kb)

Additional file 7: **Figure S3.** a qRT-PCR of SPAG5, MYCBP and c-MYC in MDA-MB-231 and MDA-MB-468 cells treated with SPAG5 overexpression and Hs-578t and BT549 cells treated with SPAG5 siRNAs. b Regression analysis identified a positive relationship between SPAG5 and MYCBP protein expression levels in breast cancer tissues. c Western blot of c-MYC expression in CHIP samples. d qRT-PCR of CDC20, CDC25C, RAD51, BRCA1 and BRCA2 in MDA-MB-231 and MDA-MB-468 cells treated with SPAG5 overexpression and Hs-578t cells treated with SPAG5 siRNAs. e qRT-PCR of CDC20, CDC25C, RAD51, BRCA1 and BRCA2 in MDA-MB-231 and MDA-MB-468 cells treated with c-MYC siRNAs. (PDF 139 kb)

Additional file 8: **Table S5.** Differential mRNA expression in SPAG5 overexpression MDA-MB-231 cells compared with that in vector cells in RNA-sequencing analysis. (XLSX 6260 kb)

Additional file 9: **Figure S4.** MYCBP knockdown inhibits tumor growth of Hs-578t cells in vitro. a CCK-8 assays showed that knockdown of MYCBP suppressed cell proliferation in Hs-578t cells. b Flow cytometry indicates that knockdown of MYCBP suppress S to G2 transition in Hs-578t cells. c Flow cytometry indicates that knockdown of MYCBP increase cell apoptosis in Hs-578t cells. (PDF 148 kb)

Additional file 10: **Figure S5.** Representative images of flow cytometry cell cycle analysis(a) showed knockdown of MYCBP or c-MYC partially reverse the increased S to G2 transition induced by overexpression of SPAG5 in MDA-MB-231 and MDA-MB-468 cells. Representative images of flow cytometry apoptosis analysis(b) showed knockdown of MYCBP or c-MYC partially reverse the reduced cell apoptosis induced by overexpression of SPAG5 in MDA-MB-231 and MDA-MB-468 cells. (PDF 222 kb)

## Abbreviations

ANTs: Adjacent noncancerous tissues
ATCC: American Type Culture Collection
BRCA: Breast cancer-associated gene
CCK8: Cell Counting Kit-8
CHIP: Chromatin immunoprecipitation
CSC: Cancer stem cell
DFS: Disease-free survival
DMSO: Dimethyl sulfoxide
GSEA: Gene set enrichment analysis
HR: Homologous recombination
IF: Immunofluorescence
IHC: Immunohistochemistry
IP: Immunoprecipitation
MYCBP: c-MYC binding protein
OS: Overall survival
PARPi: PARP inhibitor
PCR: Polymerase chain reaction
qRT–PCR: Quantitative real-time PCR
SPAG5: Sperm-associated antigen 5
TCGA: The Cancer Genome Atlas
TMA: Tissue microarray
TNBC: Triple-negative breast cancer

## References

[1] Bose S (2015). Triple-negative breast carcinoma: morphologic and molecular subtypes. Adv Anat Pathol.

[2] Kim H, Choi DH (2013). Distribution of BRCA1 and BRCA2 mutations in Asian patients with breast cancer. J Breast Cancer.

[3] Muendlein A, Rohde BH, Gasser K, Haid A, Rauch S, Kinz E, et al. Evaluation of BRCA1/2 mutational status among German and Austrian women with triple-negative breast cancer. J Cancer Res Clin. 2015. 10.1007/s00432-015-1986-2. 10.1007/s00432-015-1986-2 PMC11823898 25971625

[4] Tang H, Qiao J, Fu Y (2016). Immunotherapy and tumor microenvironment. Cancer Lett.

[5] Nanda R, Chow LQ, Dees EC, Berger R, Gupta S, Geva R, et al. Pembrolizumab in patients with advanced triple-negative breast cancer: phase Ib KEYNOTE-012 study. J Clin Oncol. 2016. 10.1200/JCO.2015.64.8931. 10.1200/JCO.2015.64.8931 PMC6816000 27138582

[6] Sharma P (2016). Biology and management of patients with triple-negative breast cancer. Oncologist.

[7] Abdel-Fatah T, Agarwal D, Liu DX, Russell R, Rueda OM, Liu K (2016). SPAG5 as a prognostic biomarker and chemotherapy sensitivity predictor in breast cancer: a retrospective, integrated genomic, transcriptomic, and protein analysis. Lancet Oncol.

[8] Liu JY, Zeng QH, Cao PG, Xie D, Yang F, He LY (2018). SPAG5 promotes proliferation and suppresses apoptosis in bladder urothelial carcinoma by upregulating Wnt3 via activating the AKT/mTOR pathway and predicts poorer survival. Oncogene.

[9] Yuan LJ, Li JD, Zhang L, Wang JH, Wan T, Zhou Y (2015). SPAG5 upregulation predicts poor prognosis in cervical cancer patients and alters sensitivity to taxol treatment via the mTOR signaling pathway. Cell Death Dis.

[10] Zhang H, Li S, Yang X, Qiao B, Zhang Z, Xu Y (2016). miR-539 inhibits prostate cancer progression by directly targeting SPAG5. J Exp Clin Cancer Res.

[11] Bertucci F, Viens P, Birnbaum D (2016). SPAG5: the ultimate marker of proliferation in early breast cancer?. Lancet Oncol.

[12] Lord CJ, Ashworth A. PARP inhibitors: synthetic lethality in the clinic. Science. 2017;355(6330):1152–8. 10.1126/science.aam7344. 10.1126/science.aam7344 PMC6175050 28302823

[13] Tahara M, Inoue T, Sato F, Miyakura Y, Horie H, Yasuda Y (2014). The use of olaparib (AZD2281) potentiates SN-38 cytotoxicity in colon cancer cells by indirect inhibition of Rad51-mediated repair of DNA double-strand breaks. Mol Cancer Ther.

[14] Kern DM, Monda JK, Su KC, Wilson-Kubalek EM, Cheeseman IM. Astrin-SKAP complex reconstitution reveals its kinetochore interaction with microtubule-bound Ndc80. Elife. 2017;6. 10.7554/eLife.26866. 10.7554/eLife.26866 PMC5602300 28841134

[15] Taira T, Maeda J, Onishi T, Kitaura H, Yoshida S, Kato H (1998). AMY-1, a novel C-MYC binding protein that stimulates transcription activity of C-MYC. Genes Cells.

[16] Chen Y, Olopade OI (2008). MYC in breast tumor progression. Expert Rev Anticancer Ther.

[17] Carey J, Karakas C, Bui T, Chen X, Vijayaraghavan S, Zhao Y (2018). Synthetic lethality of PARP inhibitors in combination with MYC blockade is independent of BRCA status in triple-negative breast cancer. Cancer Res.

[18] Li B, Severson E, Pignon JC, Zhao H, Li T, Novak J (2016). Comprehensive analyses of tumor immunity: implications for cancer immunotherapy. Genome Biol.

[19] Li M, Li A, Zhou S, Xu Y, Xiao Y, Bi R (2018). Heterogeneity of PD-L1 expression in primary tumors and paired lymph node metastases of triple negative breast cancer. BMC Cancer.

[20] Fischer M, Quaas M, Nickel A, Engeland K (2015). Indirect p53-dependent transcriptional repression of Survivin, CDC25C, and PLK1 genes requires the cyclin-dependent kinase inhibitor p21/CDKN1A and CDE/CHR promoter sites binding the DREAM complex. Oncotarget.

[21] Yang YF, Zhang MF, Tian QH, Fu J, Yang X, Zhang CZ (2018). SPAG5 interacts with CEP55 and exerts oncogenic activities via PI3K/AKT pathway in hepatocellular carcinoma. Mol Cancer.

[22] Thedieck K, Holzwarth B, Prentzell MT, Boehlke C, Klasener K, Ruf S (2013). Inhibition of mTORC1 by astrin and stress granules prevents apoptosis in cancer cells. Cell.

[23] Saldivar JC, Hamperl S, Bocek MJ, Chung M, Bass TE, Cisneros-Soberanis F (2018). An intrinsic S/G2 checkpoint enforced by ATR. Science.

[24] Shah AP, Patel CN, Sureja DK, Sanghavi KP (2018). A review on DNA repair inhibition by PARP inhibitors in cancer therapy. Folia Med.

[25] Tsibulak I, Wieser V, Degasper C, Shivalingaiah G, Wenzel S, Sprung S, et al. BRCA1 and BRCA2 mRNA-expression prove to be of clinical impact in ovarian cancer. Br J Cancer. 2018. 10.1038/s41416-018-0217-4. 10.1038/s41416-018-0217-4 PMC6173779 30111871

[26] Xiong J, Du Q, Liang Z (2010). Tumor-suppressive microRNA-22 inhibits the transcription of E-box-containing c-Myc target genes by silencing c-Myc binding protein. Oncogene.

[27] Jiang X, Hu C, Arnovitz S, Bugno J, Yu M, Zuo Z (2016). miR-22 has a potent anti-tumour role with therapeutic potential in acute myeloid leukaemia. Nat Commun.

[28] Gong L, Xia Y, Qian Z, Shi J, Luo J, Song G (2018). Overexpression of MYC binding protein promotes invasion and migration in gastric cancer. Oncol Lett.

[29] Fallah Y, Brundage J, Allegakoen P, Shajahan-Haq AN (2017). MYC-driven pathways in breast cancer subtypes. Biomolecules.

[30] Hynes NE, Stoelzle T (2009). Key signalling nodes in mammary gland development and cancer: Myc. Breast Cancer Res.

[31] Wang Z, Yang B, Zhang M, Guo W, Wu Z, Wang Y (2018). lncRNA epigenetic landscape analysis identifies EPIC1 as an oncogenic lncRNA that interacts with MYC and promotes cell-cycle progression in cancer. Cancer Cell.

[32] Mao DY, Watson JD, Yan PS, Barsyte-Lovejoy D, Khosravi F, Wong WW (2003). Analysis of Myc bound loci identified by CpG island arrays shows that max is essential for Myc-dependent repression. Curr Biol.

[33] Luoto KR, Meng AX, Wasylishen AR, Zhao H, Coackley CL, Penn LZ (2010). Tumor cell kill by c-MYC depletion: role of MYC-regulated genes that control DNA double-strand break repair. Cancer Res.

