# Supplementary figures and images for: SPAG5 upregulation contributes to enhanced c-MYC transcriptional activity via interaction with c-MYC binding protein in triple-negative breast cancer
Source: J Hematol Oncol. 2019 Feb 8;12:14. doi: 10.1186/s13045-019-0700-2 (PMC6367803; doi:10.1186/s13045-019-0700-2)

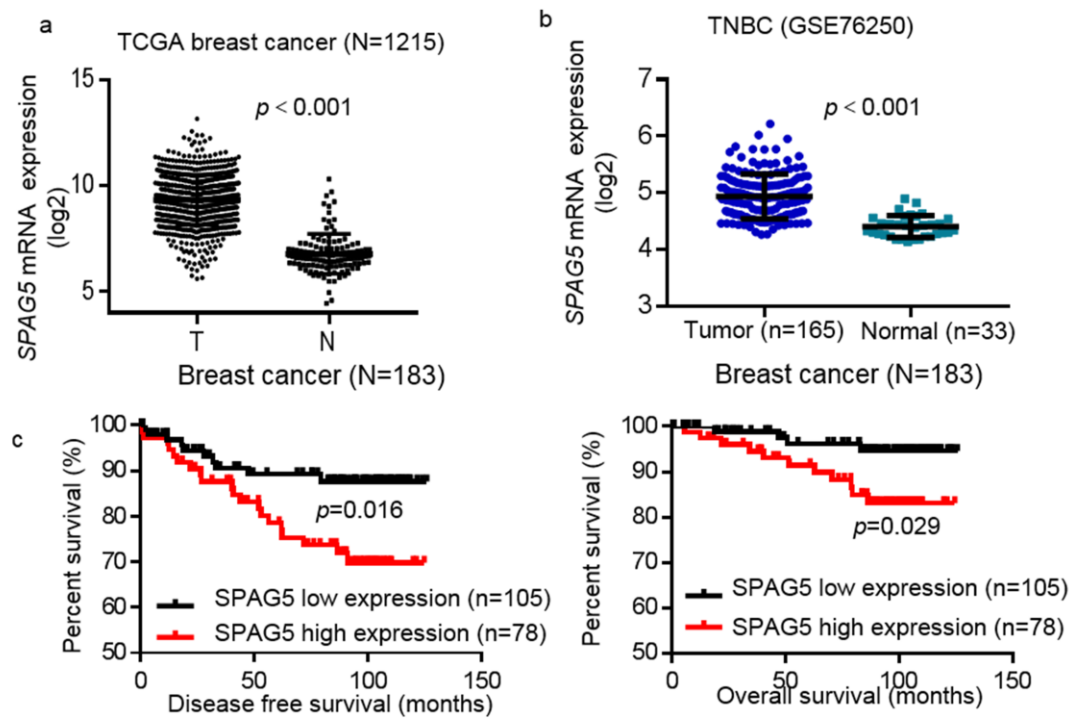

Supplement: Supplementary file 2 — Figure S1. Increased SPAG5 expression promotes breast cancer progression and correlates with poor prognosis. a SPAG5 mRNA levels in TCGA breast cancer mRNA dataset of tumor(n = 737) versus non-tumor tissues(n = 120). b SPAG5 mRNA levels in TNBC (n = 165) versus non-tumor tissues(n = 33) from GSE76250 dataset. c Kaplan–Meier curve of DFS and OS for breast cancer patients with low expression of SPAG5 versus high expression of SPAG5 group. (PDF 267 kb) [file 13045_2019_700_MOESM2_ESM.pdf]

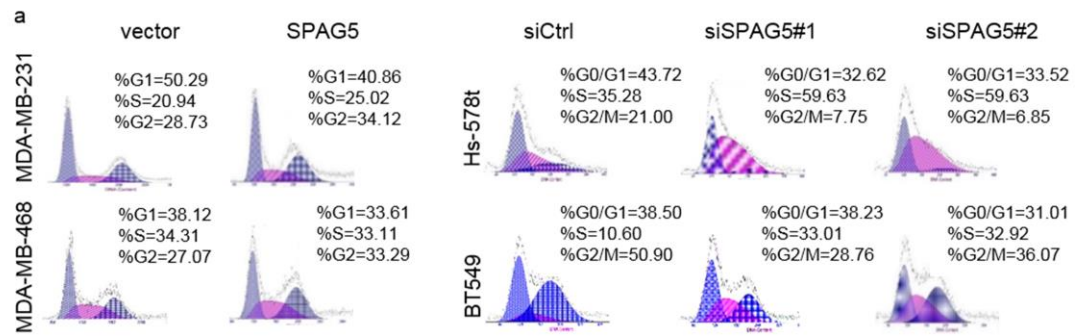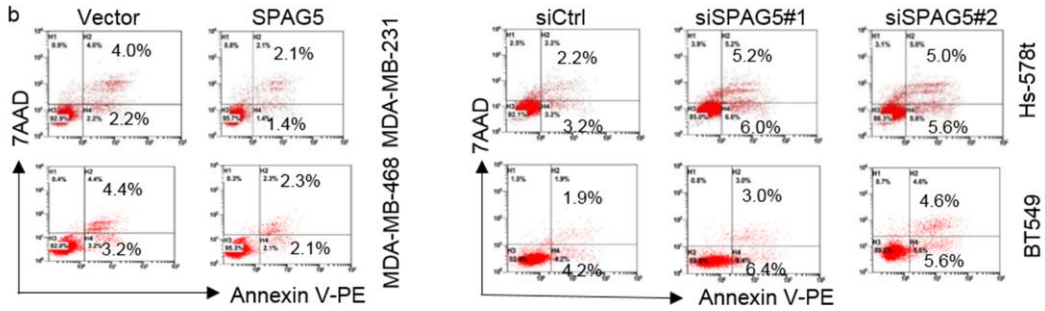

Supplement: Supplementary file 6 — Figure S2. Representative images of flow cytometry cell cycle analysis(a) and apoptosis analysis(b) in MDA-MB-231, MDA-MB-468 cells, Hs-578t and BT549 cells. (PDF 267 kb) (PDF 148 kb) [file 13045_2019_700_MOESM6_ESM.pdf]

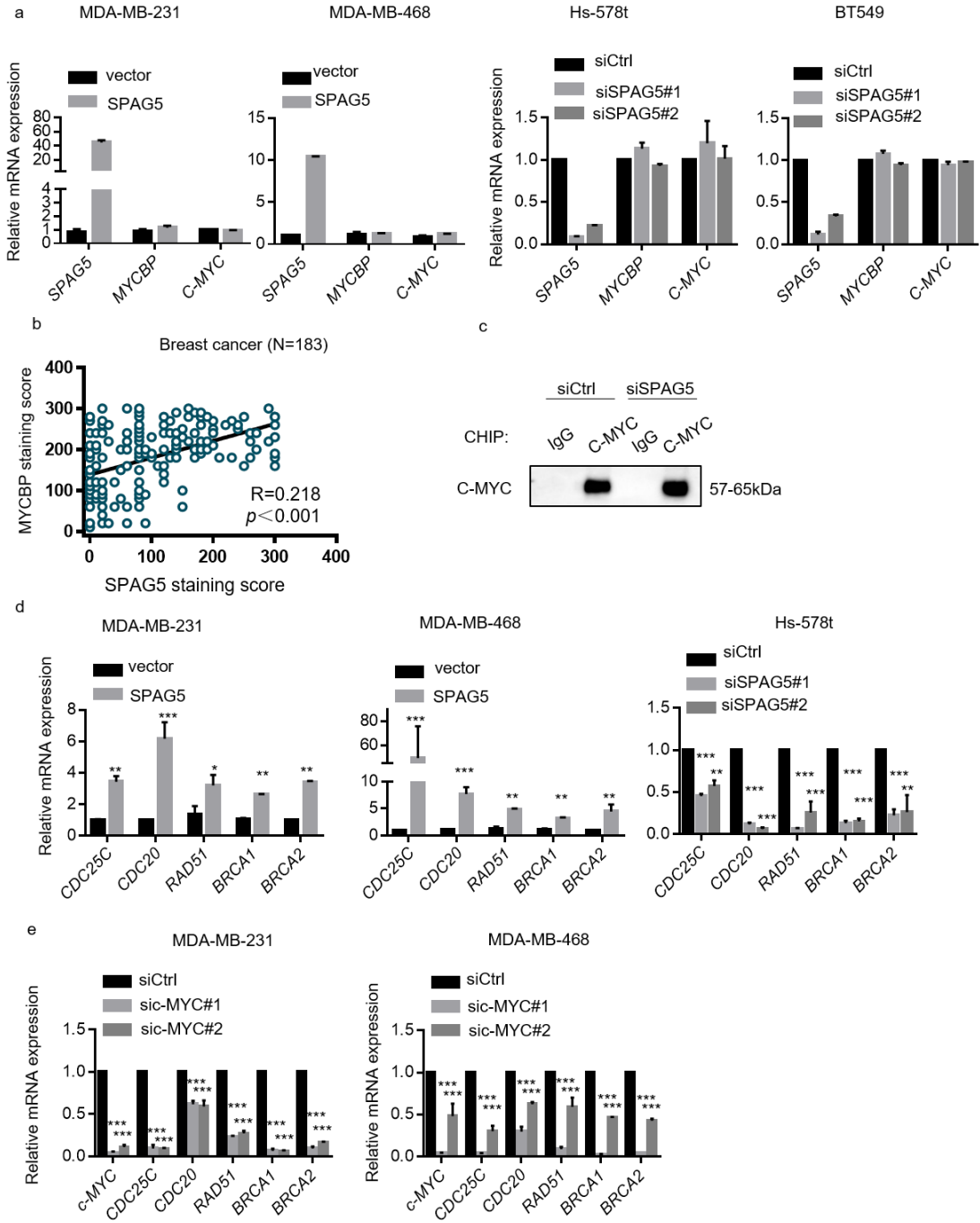

Supplement: Supplementary file 7 — Figure S3. a qRT-PCR of SPAG5, MYCBP and c-MYC in MDA-MB-231 and MDA-MB-468 cells treated with SPAG5 overexpression and Hs-578t and BT549 cells treated with SPAG5 siRNAs. b Regression analysis identified a positive relationship between SPAG5 and MYCBP protein expression levels in breast cancer tissues. c Western blot of c-MYC expression in CHIP samples. d qRT-PCR of CDC20, CDC25C, RAD51, BRCA1 and BRCA2 in MDA-MB-231 and MDA-MB-468 cells treated with SPAG5 overexpression and Hs-578t cells treated with SPAG5 siRNAs. e qRT-PCR of CDC20, CDC25C, RAD51, BRCA1 and BRCA2 in MDA-MB-231 and MDA-MB-468 cells treated with c-MYC siRNAs. (PDF 139 kb) [file 13045_2019_700_MOESM7_ESM.pdf]

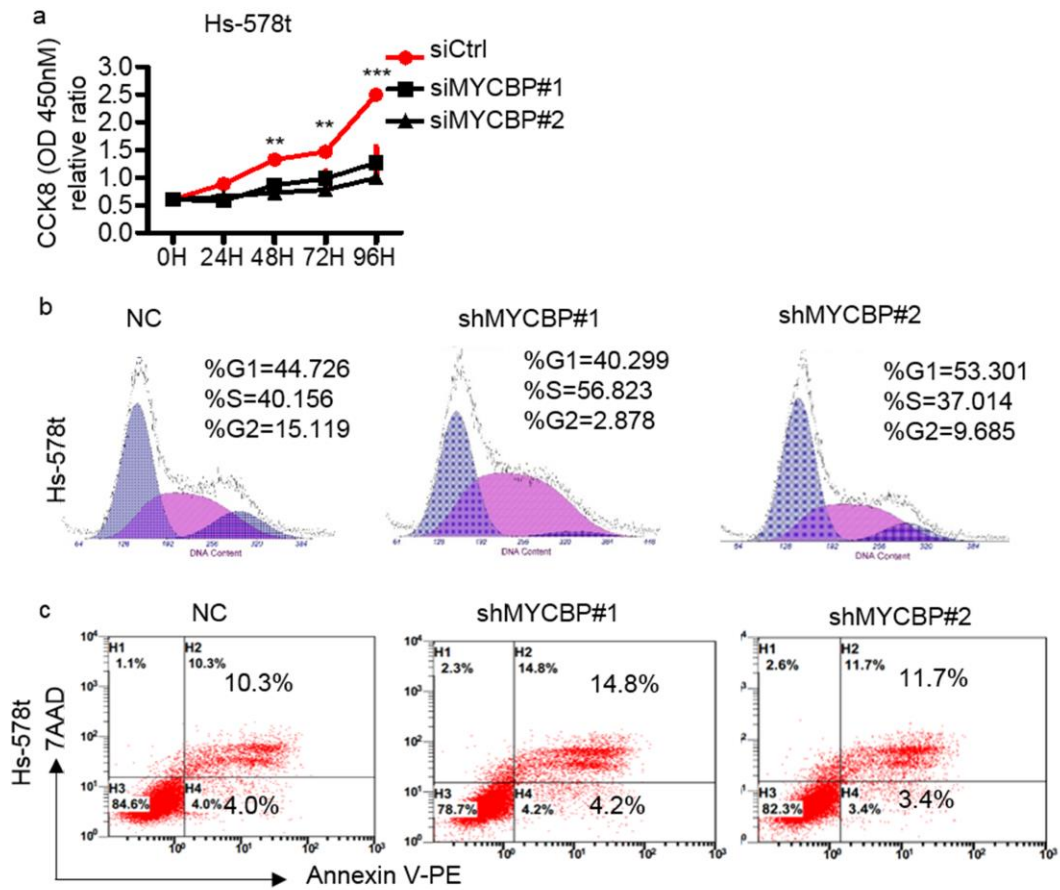

Supplement: Supplementary file 9 — Figure S4. MYCBP knockdown inhibits tumor growth of Hs-578t cells in vitro. a CCK-8 assays showed that knockdown of MYCBP suppressed cell proliferation in Hs-578t cells. b Flow cytometry indicates that knockdown of MYCBP suppress S to G2 transition in Hs-578t cells. c Flow cytometry indicates that knockdown of MYCBP increase cell apoptosis in Hs-578t cells. (PDF 148 kb) [file 13045_2019_700_MOESM9_ESM.pdf]

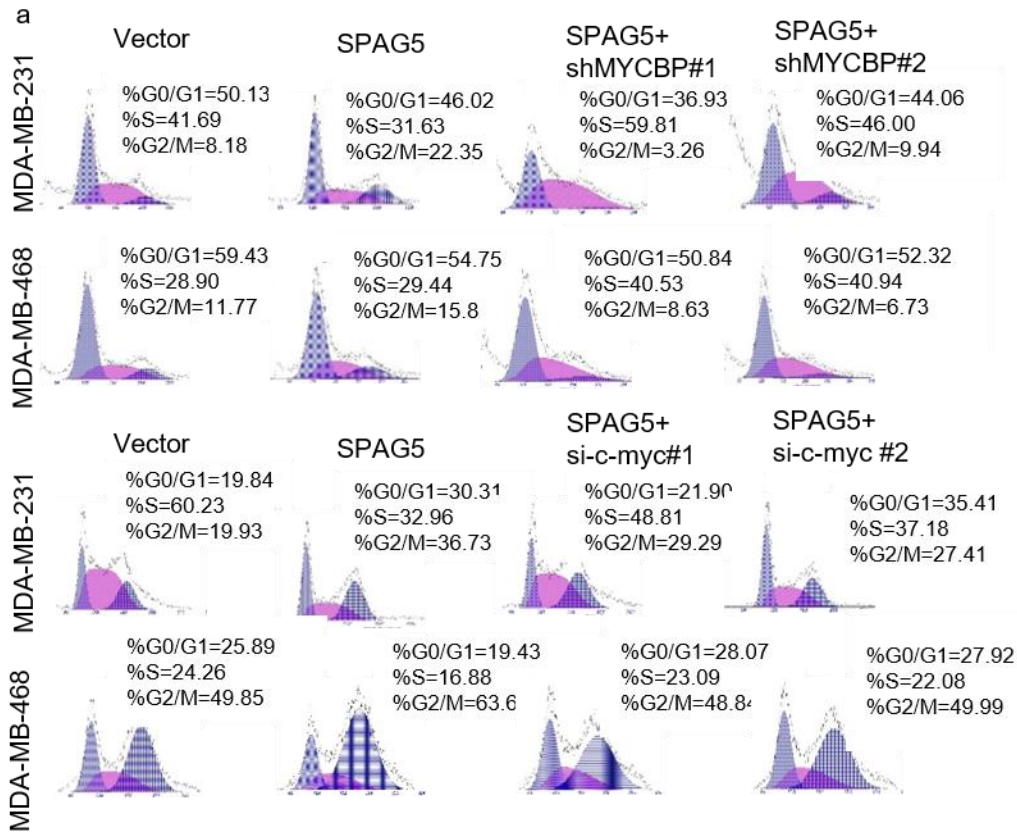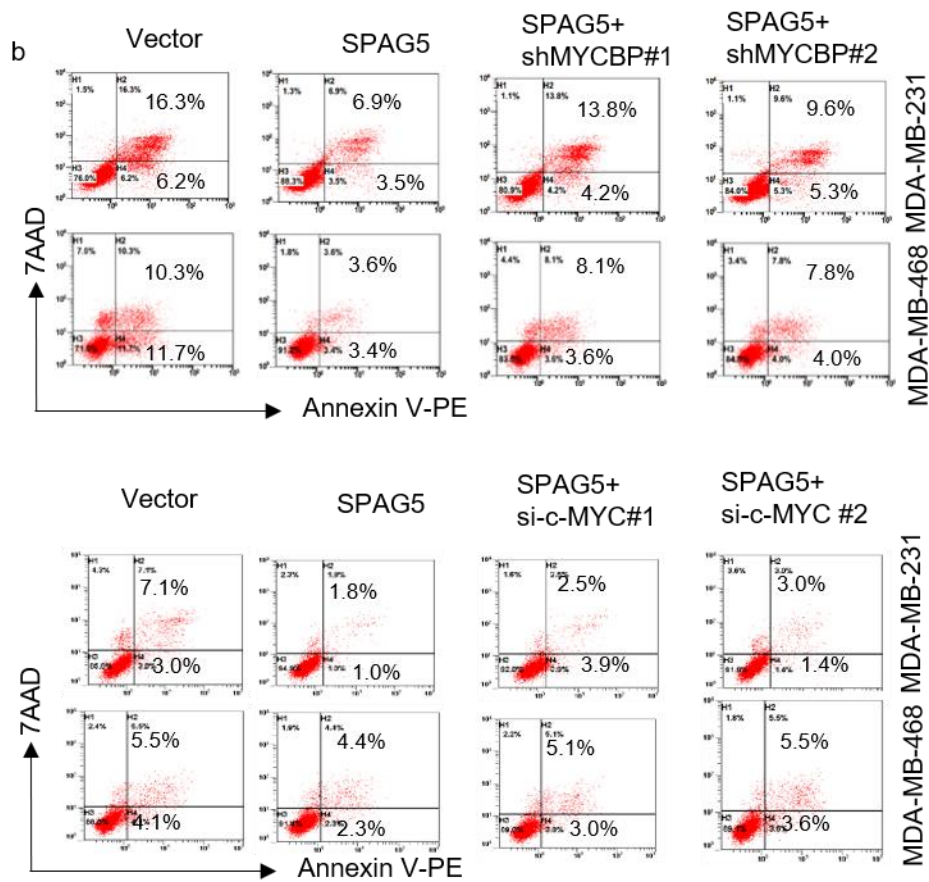

Supplement: Supplementary file 10 — Figure S5. Representative images of flow cytometry cell cycle analysis(a) showed knockdown of MYCBP or c-MYC partially reverse the increased S to G2 transition induced by overexpression of SPAG5 in MDA-MB-231 and MDA-MB-468 cells. Representative images of flow cytometry apoptosis analysis(b) showed knockdown of MYCBP or c-MYC partially reverse the reduced cell apoptosis induced by overexpression of SPAG5 in MDA-MB-231 and MDA-MB-468 cells. (PDF 222 kb) [file 13045_2019_700_MOESM10_ESM.pdf]
