# Supplementary material for: SPAG5 upregulation contributes to enhanced c-MYC transcriptional activity via interaction with c-MYC binding protein in triple-negative breast cancer
Source: J Hematol Oncol. 2019 Feb 8;12:14. doi: 10.1186/s13045-019-0700-2 (PMC6367803; doi:10.1186/s13045-019-0700-2)
Supplement: Supplementary file 3 — Table S2. Correlation between SPAG5 expression and clinical features of breast cancer patients. (DOCX 35 kb) [file 13045_2019_700_MOESM3_ESM.docx]

**Table S2** Correlation of SPAG5 expression and clinical features of breast cancer patients

| Variable | Overall (N=183) | | SPAG5 | | | | |
| --- | --- | --- | --- | --- | --- | --- | --- |
|  |  |  | Low expression (N=105) | | High expression (N=78) | |  |
|  | N | % | N | % | N | % | *P* |
| **Age, years** |  |  |  |  |  |  | 0.359 |
| ≤50 | 101 | 55.19 | 61 | 58.10 | 40 | 51.28 |  |
| >50 | 82 | 44.81 | 44 | 41.90 | 38 | 48.72 |  |
| **Tumor size, cm** |  |  |  |  |  |  | 0.501 |
| <2 | 87 | 47.54 | 49 | 46.67 | 38 | 48.72 |  |
| 2≤T<5 | 87 | 47.54 | 49 | 46.67 | 38 | 48.72 |  |
| ≥5 | 9 | 4.92 | 7 | 6.67 | 2 | 2.56 |  |
| **Histological grade** |  |  |  |  |  |  | 0.93 |
| I/II | 132 | 72.13 | 76 | 72.38 | 56 | 71.79 |  |
| III | 51 | 27.87 | 29 | 27.62 | 22 | 28.21 |  |
| **Node status** |  |  |  |  |  |  | 0.648 |
| pN0 (none) | 89 | 48.63 | 48 | 45.71 | 41 | 52.56 |  |
| pN1 (1-3) | 50 | 27.32 | 29 | 27.62 | 21 | 26.92 |  |
| pN2 (4-9) | 17 | 9.29 | 12 | 11.43 | 5 | 6.41 |  |
| pN3 (≥10) | 25 | 13.66 | 14 | 13.33 | 11 | 14.10 |  |
| pNX | 2 | 1.09 | 2 | 1.90 | 0 | 0.00 |  |
| **Molecular subtype** |  |  |  |  |  |  | 0.251 |
| Luminal A | 48 | 26.23 | 32 | 30.48 | 16 | 20.51 |  |
| Luminal B | 44 | 24.04 | 23 | 21.90 | 21 | 26.92 |  |
| Her2 subtype | 49 | 26.78 | 30 | 28.57 | 19 | 24.36 |  |
| TNBC | 42 | 22.95 | 20 | 19.05 | 22 | 28.21 |  |
| **Local recurrence** |  |  |  |  |  |  | **＜0.001** |
| absence | 174 | 95.08 | 105 | 100.00 | 69 | 88.46 |  |
| presence | 9 | 4.92 | 0 | 0.00 | 9 | 11.54 |  |
| **Distant Metastasis** |  |  |  |  |  |  | 0.337 |
| absence | 162 | 88.52 | 95 | 90.48 | 67 | 85.90 |  |
| presence | 21 | 11.48 | 10 | 9.52 | 11 | 14.10 |  |
