# Supplementary material for: SPAG5 upregulation contributes to enhanced c-MYC transcriptional activity via interaction with c-MYC binding protein in triple-negative breast cancer
Source: J Hematol Oncol. 2019 Feb 8;12:14. doi: 10.1186/s13045-019-0700-2 (PMC6367803; doi:10.1186/s13045-019-0700-2)
Supplement: Supplementary file 4 — Table S3. Univariate and multivariate analyses of SPAG5 expression and prognosis in breast cancer patients. (DOCX 24 kb) [file 13045_2019_700_MOESM4_ESM.docx]

**Table S3** Univariate and multivariate analyses of SPAG5 expression and prognosis in breast cancer patients

| Variable | DFS | | | | | | OS | | | | | |
| --- | --- | --- | --- | --- | --- | --- | --- | --- | --- | --- | --- | --- |
|  | Univariate analysis | | | Multivariate analysis | | | Univariate analysis | | | Multivariate analysis | | |
|  | HR | 95% CI | *P* | HR | 95% CI | *P* | HR | 95% CI | *P* | HR | 95% CI | *P* |
| SPAG5 | 2.47 | 1.203-5.073 | ***0.016*** | 2.5 | 1.176-5.312 | ***0.017*** | 3.327 | 1.204-9.196 | ***0.029*** | 3.327 | 1.059-10.450 | ***0.04*** |
| Age | 0.494 | 0.632-2.588 | 0.494 |  |  |  | 2.127 | 0.756-5.981 | 0.153 |  |  |  |
| Tumor size | 1.008 | 0.534-1.905 | 0.98 |  |  |  | 1.459 | 0.586-3.635 | 0.417 |  |  |  |
| Histological grade | 1.275 | 0.619-2.625 | 0.51 |  |  |  | 1.643 | 0.600-4.503 | 0.334 |  |  |  |
| Node status | 1.574 | 0.771-3.212 | 0.213 |  |  |  | 2.175 | 0.743-6.364 | 0.156 |  |  |  |
| Molecular subtype | 1.402 | 1.019-1.930 | ***0.038*** | 1.378 | 0.997-1.904 | 0.052 | 1.277 | 0.818-1.995 | 0.282 |  |  |  |
