# Supplementary material for: SPAG5 upregulation contributes to enhanced c-MYC transcriptional activity via interaction with c-MYC binding protein in triple-negative breast cancer
Source: J Hematol Oncol. 2019 Feb 8;12:14. doi: 10.1186/s13045-019-0700-2 (PMC6367803; doi:10.1186/s13045-019-0700-2)
Supplement: Supplementary file 5 — Table S4. Univariate and multivariate analyses of SPAG5 expression and OS in TNBC patients. (DOCX 19 kb) [file 13045_2019_700_MOESM5_ESM.docx]

**Table S4** Univariate and multivariate analyses of SPAG5 expression and OS in TNBC patients

| Variable | OS | | | | | |
| --- | --- | --- | --- | --- | --- | --- |
|  | Univariate analysis | | | Multivariate analysis | | |
|  | HR | 95% CI | *P* | HR | 95% CI | *P* |
| SPAG5 | 6.279 | 0.755-52.205 | ***0.084*** |  |  |  |
| Age | 2.466 | 0.478-12.733 | 0.281 |  |  |  |
| Tumor size | 1.398 | 0.400-4.890 | 0.600 |  |  |  |
| Histological grade | 1.734 | 0.414-7.261 | 0.451 |  |  |  |
| Node status | 7.989 | 0.961-66.435 | 0.055 |  |  |  |
